# Recent Trends in the Synthesis and Bioactivity of Coumarin, Coumarin–Chalcone, and Coumarin–Triazole Molecular Hybrids

**DOI:** 10.3390/molecules29051026

**Published:** 2024-02-27

**Authors:** Nur Rohman, Bayu Ardiansah, Tuti Wukirsari, Zaher Judeh

**Affiliations:** 1 Department of Chemistry, Faculty of Mathematics and Natural Sciences, Universitas Indonesia, Depok 16424, Indonesia; nur.rohman@ui.ac.id (N.R.); tutiwukirsari@sci.ui.ac.id (T.W.); 2 School of Chemistry, Chemical Engineering and Biotechnology, Nanyang Technological University, 62 Nanyang Drive, N1.2-B1-14, Singapore 637459, Singapore

**Keywords:** molecular hybridization, biological activity, coumarin, chalcone, triazole

## Abstract

Molecular hybridization represents a new approach in drug discovery in which specific chromophores are strategically combined to create novel drugs with enhanced therapeutic effects. This innovative strategy leverages the strengths of individual chromophores to address complex biological challenges, synergize beneficial properties, optimize pharmacokinetics, and overcome limitations associated with single-agent therapies. Coumarins are documented to possess several bioactivities and have therefore been targeted for combination with other active moieties to create molecular hybrids. This review summarizes recent (2013–2023) trends in the synthesis of coumarins, as well as coumarin–chalcone and coumarin–triazole molecular hybrids. To cover the wide aspects of this area, we have included differently substituted coumarins, chalcones, 1,2,3– and 1,2,4–triazoles in this review and considered the point of fusion/attachment with coumarin to show the diversity of these hybrids. The reported syntheses mainly relied on well-established chemistry without the need for strict reaction conditions and usually produced high yields. Additionally, we discussed the bioactivities of the reported compounds, including antioxidative, antimicrobial, anticancer, antidiabetic, and anti-cholinesterase activities and commented on their IC_50_ where possible. Promising bioactivity results have been obtained so far. It is noted that mechanistic studies are infrequently found in the published work, which was also mentioned in this review to give the reader a better understanding. This review aims to provide valuable information to enable further developments in this field.

## 1. Introduction

Molecular hybridization (MH) is an established drug design method used by medicinal chemists to create and refine new lead compounds [1]. It is the rational combination of two or more pharmacophoric units with distinct modes of action into a single molecule [2,3]. MH is expected to increase the biological activity of newly created pharmacophores [4] through alterations to their pharmacokinetic profiles, modes of action, potency, selectivity, and biological activity [5].

**Figure 1:**
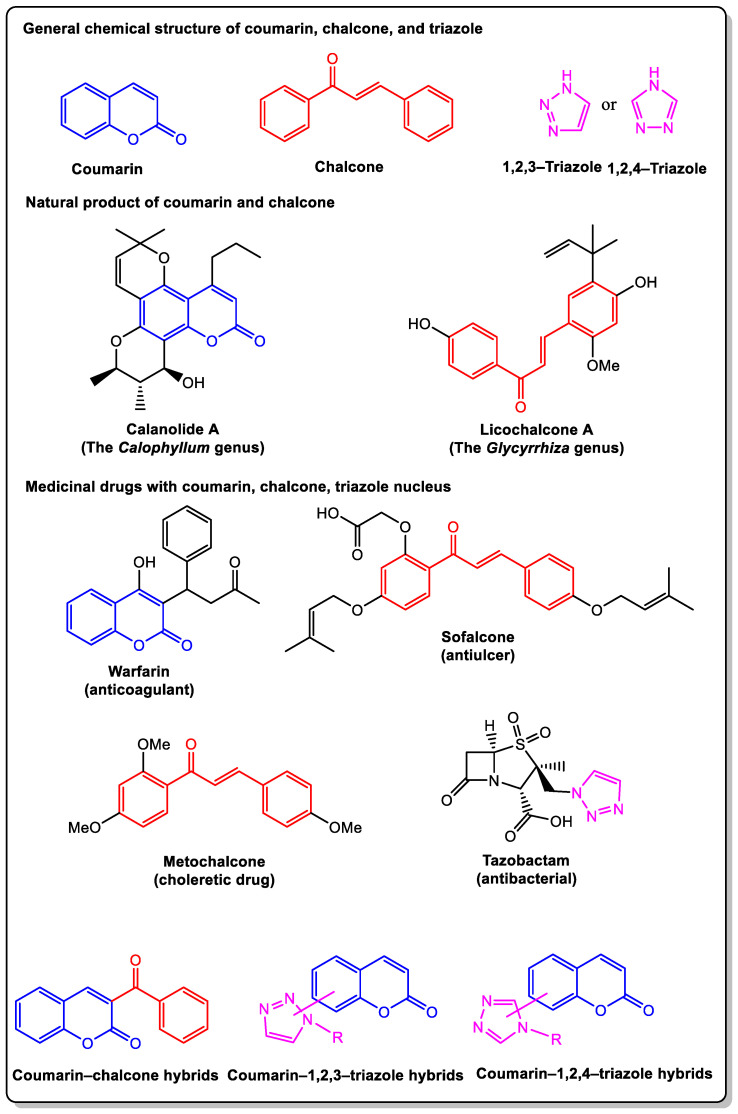
Structures of coumarin, chalcone and triazole and their derivatives.

**Figure 2:**
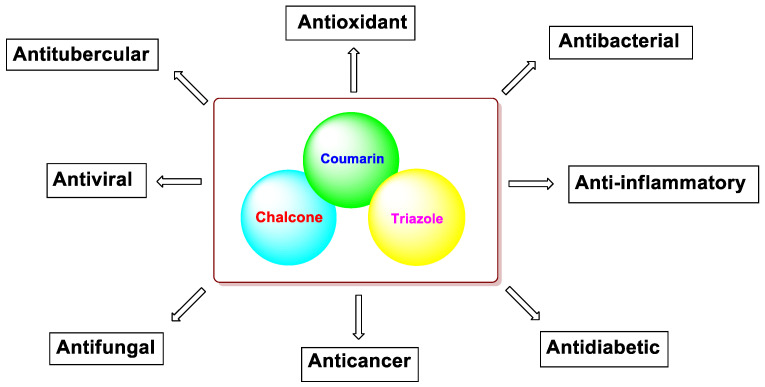
Significant biological activities of coumarin, chalcone and triazole derivatives.

Coumarins are a class of naturally occurring *O*-heterocyclic compounds with 2*H*-1-benzopyran-2-one as a core framework (Figure 1) [6,7] Coumarins are widely recognized for their pharmacological effects in both natural and synthetic forms. They possess antifungal, antibacterial, anti-inflammatory, antioxidant, anti-cholinesterase, antidiabetic, antineoplastic, anti-HIV, anticoagulant, and anticancer bioactivities (Figure 2) [8,9,10,11,12,13,14,15]. For instance, the naturally occurring coumarin Calanolide A (Figure 1), which was extracted from the species *Calophyllum*, showed interesting anti-HIV activity [16]. Warfarin is a synthetic anticoagulant drug that blocks the formation of blood clots [17]. Due to their important bioactivity, more research has recently focused on how coumarins might be combined with other chromophores to create new structural diversity with improved activity.

Chalcones (1,3-diaryl-2-propen-1-ones) (Figure 1) are made up of two aromatic rings linked by an α,β-unsaturated carbonyl group [18,19,20]. Researchers have expressed interest in the chalcone scaffold, and several studies have emphasized the significance of these molecules in the domains of pharmacology and chemistry [21]. Because of their significant antioxidant, antiviral, antidiabetic, anticancer, anti-inflammatory, antibacterial, antifungal, and antihyperlipidemic properties, chalcones, both natural and synthetic, are of great chemical and pharmacological interest (Figure 2) [22,23,24,25,26,27,28,29,30,31,32]. For example, Licochalcone A is a naturally occurring chalcone isolated from the roots of *Glycyrrhiza* species, specifically *G. glabra* and *inflata*. It possesses anti-inflammatory, anticancer and antioxidant properties (Figure 1) [33,34,35,36,37]. Several chalcone-based drugs have been licensed for use in therapeutic settings. For instance, sofalcone is used as an antiulcer and mucoprotective medication, while metochalcone is used as a choleretic drug (Figure 1) [38,39,40].

Triazoles are heterocyclic compounds with a basic framework comprising a five-membered ring containing three nitrogen atoms at positions 1,2,3- or 1,2,4 in the ring. (Figure 1). Because of their excellent chemical stability against oxidation, reduction, and hydrolysis, they have a wide range of medical applications [41,42,43,44]. Due to their wide range of biological significance, triazoles have been recognized as prominent pharmacophores for several commercial drugs (Figure 2) [45,46,47,48,49,50,51,52,53]. For example, tazobactam (Figure 1) is an antibacterial drug that treats infections in both adult and pediatric patients [54,55,56,57].

Coumarin derivatives are popular and attractive in the field of medicinal/organic chemistry [58,59,60,61,62,63,64,65,66,67,68]. Hence, several review papers on this topic have been published. For example, Adimule et al. (2022) arranged a recent advance in the one-pot synthesis of coumarin derivatives from different starting materials using metal nanoparticles as heterogeneous catalysts [7]. Song et al. (2020) released an update on coumarin derivatives with anticancer activities by underlining interactions between coumarin derivatives and diverse enzymes and receptors in cancer cells [11]. Additionally, the recent development of coumarin hybrids as antifungal agents was summarized by Hu et al. [14]. Furthermore, an overview of synthetic approaches and the biological activity of coumarin derivatives was provided by Annunziata et al. [67]. The present review focuses on coumarin hybrids. However, it starts with recent developments in coumarin chemistry to give the reader the necessary background and transition needed for the chemistry of coumarin hybrids. We attempt to summarize the synthesis strategies and biological activities of coumarin, coumarin–chalcone and coumarin–triazole hybrids. This review will be beneficial for synthetic and medicinal chemists aiming to develop these hybrids into lead drug compounds.

## 2. Synthesis of Coumarin, Coumarin–Chalcone Hybrids, and Coumarin–Triazole Hybrids

### 2.1. Synthesis of Coumarin Derivatives

**Scheme 1:**
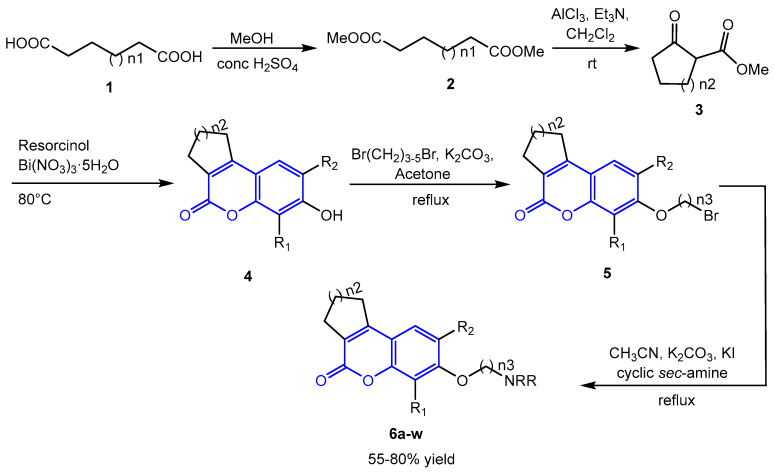
Synthesis of coumarins **6a**–**w** from dicarboxylic acid **1** [68].

Chen et al. (2014) synthesized several coumarin derivatives starting from dicarboxylic acids **1** (Scheme 1) [68]. Acid **1** was esterified to form ester **2**, which underwent cyclization to form 2-carboalkoxcyclohexanone **3**. The Pechmann reaction of **3** with resorcinol catalyzed by Bi(NO_3_)_3_ afforded compound **4**, which was bromoalkylated to form **5**. Compound **5** was reacted with various cyclic secondary amines to give amino-functionalized coumarins (**6a**–**w**) in medium-to-good yields.

**Scheme 2:**
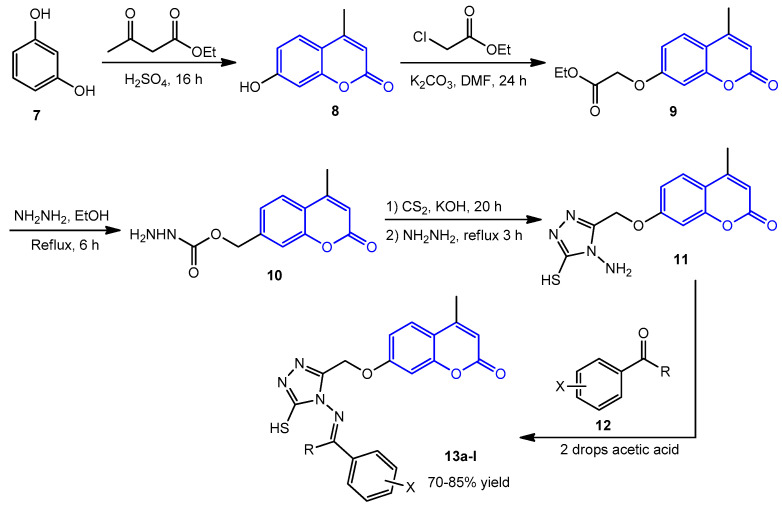
Synthesis of coumarins **13a**–**l** from resorcinol **7** [69].

Elbastawesy et al. (2015) constructed twelve novel coumarin derivates, starting from resorcinol **7** (Scheme 2) [69]. After subsequent cyclization, following an S_N_2 reaction with ethyl chloroacetate and then amidation with hydrazine, compound **10** was obtained which, after further reactions, gave compound **11**. From this important intermediate, coumarins **13a**–**l** were obtained in good yields.

**Scheme 3:**
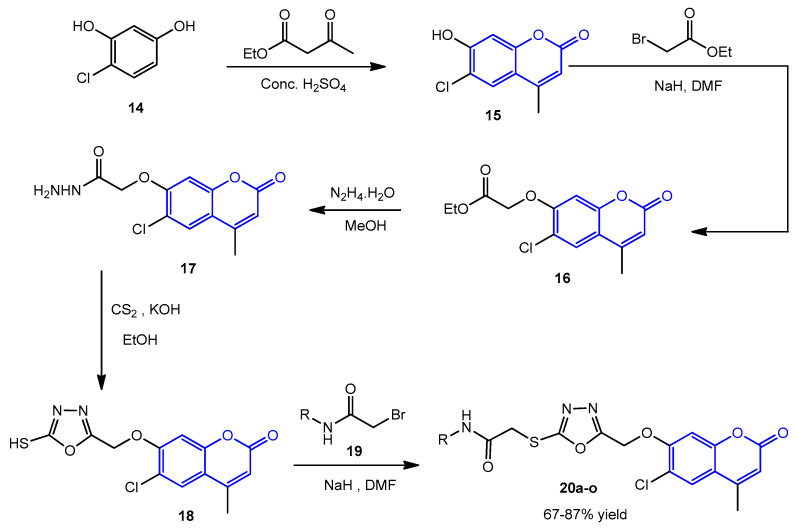
Synthesis of coumarins **20a**–**o** from 4-chlororesorcinol **14** [70].

Rasool et al. (2016) converted 4-chlororesorcinol **14** via Pechmann condensation into coumarins **20a**–**o** (Scheme 3) [70]. After Pechmann cyclization, an S_N_2 reaction of **15** with ethyl 2-bromoacetate, and then amidation, compound **17** was obtained. The derivatization of **17** gave compound **18**, which then underwent an S_N_2 reaction to give coumarins **20a**–**o** in medium-to-good yields.

**Scheme 4:**
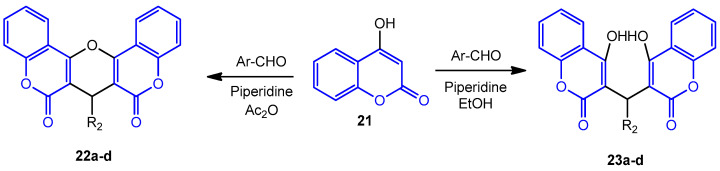
Synthesis of coumarins **22a**–**d** and **23a**–**d** from 4-hydroxycoumarin **22** [71].

Weng and Yuan (2018) successfully synthesized two types of coumarin (Scheme 4) [71]. 4-Hydroxycoumarin **21** was reacted with aromatic aldehydes via two different conditions to produce compounds **22a**–**d** and **23a**–**d**.

**Scheme 5:**
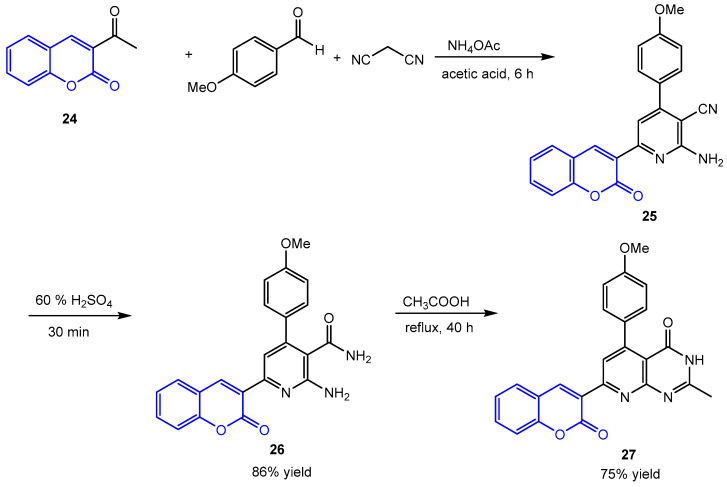
Synthesis of coumarin **27** from acetyl-2H-chromen-2-one **24** [72].

Fayed et al. (2019) synthesized coumarin derivatives through a one-pot reaction of compound **24**, 4-methoxybenzaldehyde, and malononitrile to produce intermediate **25** which, upon hydrolysis using 60% H_2_SO_4_, gave compound **26** (Scheme 5) [72]. The subsequent cyclization of **26** with glacial acetic acid gave compound **27** in a 75% yield (Scheme 5).

**Scheme 6:**
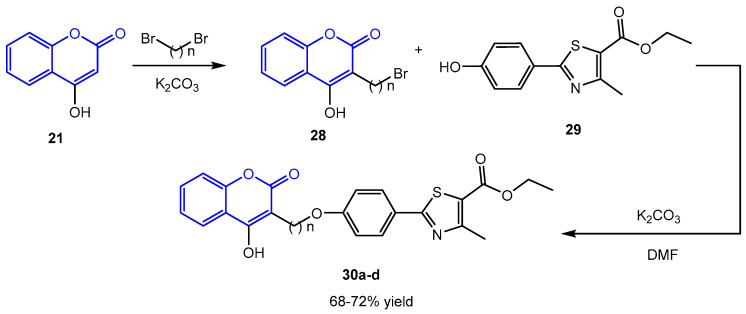
Synthesis of coumarins **30a**–**d** from 4-hydroxy coumarin **21** [73].

Naik et al. (2019) synthesized four novel coumarin derivatives (Scheme 6) [73]. An S_N_2 reaction between 4-hydroxycoumarin **21** and dibromoalkane gave intermediate **28** which, upon another S_N_2 reaction with **29**, provided coumarins **30a**–**d** in medium yields.

**Scheme 7:**
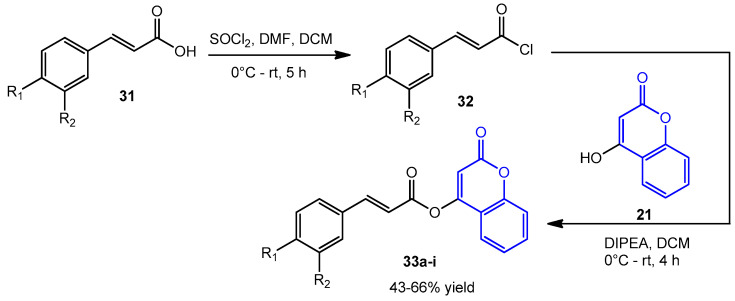
Synthesis of coumarins **33a**–**g** from substituted carboxylic acid **31** [74].

Xu et al. (2020) converted carboxylic acid **31** into cinnamoyl chlorides **32** which, upon reaction with compound **21**, gave coumarin derivatives **33a**–**i** in medium yields (Scheme 7) [74].

**Scheme 8:**
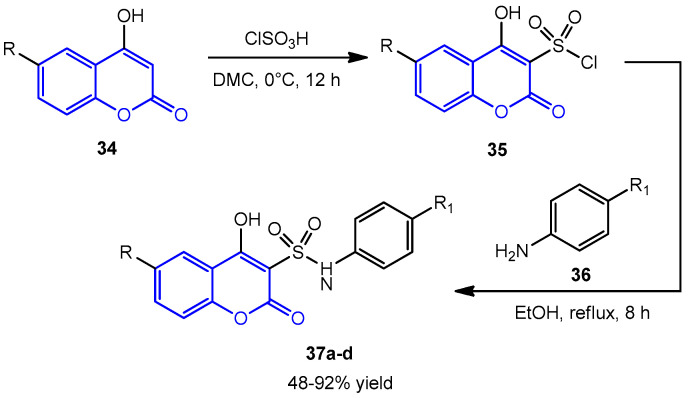
Synthesis of coumarins **37a**–**d** from 4-hydroxy-6-substituted coumarin **34** [75].

Alshibl et al. (2020) synthesized coumarin derivates via Knoevenagel condensation between **34** and chlorosulfonic acid to produce coumarin sulfonyl chloride **35**, which underwent an S_N_2 reaction with *p*-substituted aniline **36** to give coumarin-3-sulfonamides **37a**–**d** in medium-to-excellent yields (Scheme 8) [75].

**Scheme 9:**
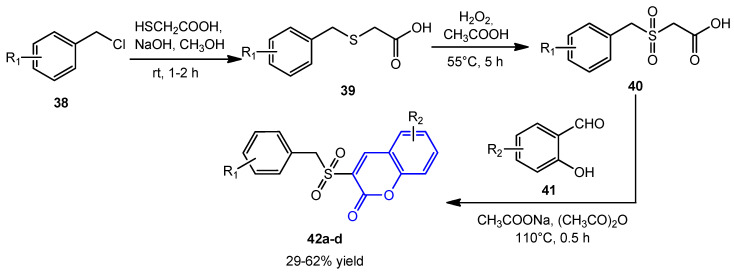
Synthesis of coumarins **42a**–**d** from substituted benzylchlorides **38** [76].

In line with the development of coumarin derivatives, Wang et al. (2020) synthesized 3-substituted coumarin derivatives via a three-step synthetic route from substituted benzyl chlorides/bromides **38** (Scheme 9) [76]. The starting materials **38** were treated with mercaptoacetic acid in the presence of sodium hydroxide to give benzylmercaptoacetic acid **39** which, upon treatment with hydrogen peroxide, gave benzylsulfonylacetic acid **40**. Finally, the target compounds **42a**–**d** were synthesized via a Knoevenagel reaction between **40** and substituted salicylaldehydes **41** and were obtained in low-to-medium yields.

**Scheme 10:**
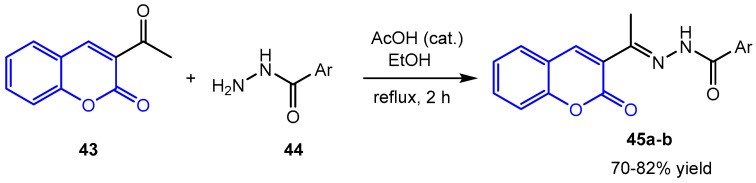
Synthesis of coumarins **45a**–**b** from 3-acetyl coumarin **43** [77].

Abduljabbar and Hadi (2021) synthesized coumarin derivatives **45a**–**b** via the acetic acid-catalyzed reaction between 3-acetyl coumarin **43** and hydrazides **44** (Scheme 10) [77].

**Scheme 11:**
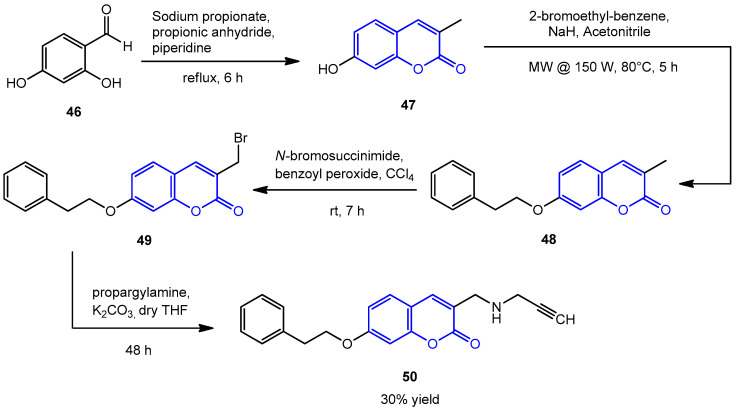
Synthesis of coumarin **50** from dihydroxybenzaldehyde **46** [78].

Mzezewa et al. (2021) initiated the synthesis of 3,7-substituted coumarin with a Pechmann condensation of dihydroxybenzaldehyde **46** to give compound **47**, which, upon esterification with 2-bromoethylbenzene, gave **48** (Scheme 11) [78]. The subsequent bromination of **48** with *N*-bromosuccinimide gave **49** which, upon reaction with propargylamine, gave coumarin **50**. 

**Scheme 12:**
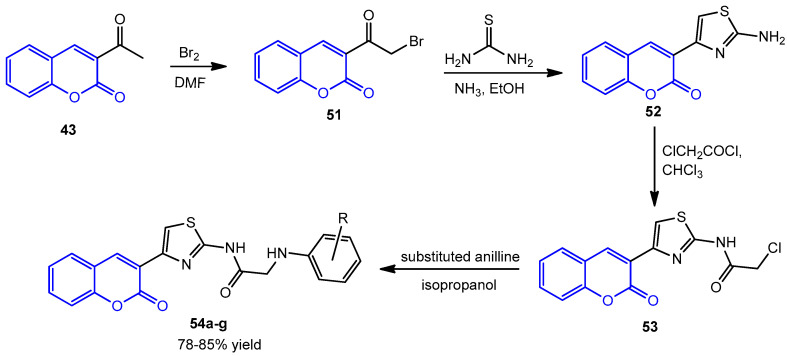
Synthesis of coumarins **54a**–**g** from 3-acetyl coumarin **43** [79].

Kokat and Jadhav (2022) constructed coumarin derivatives **54a**–**g** (Scheme 12) [79]. The bromination of 3-acetyl coumarin **43** gave **51**, which, upon cyclization with thiourea and then an S_N_2 reaction on the amino group, gave **53**. Finally, an S_N_2 reaction between substituted anilines and compound **53** resulted in the formation of the target coumarins **54a**–**g** in good yields.

**Scheme 13:**
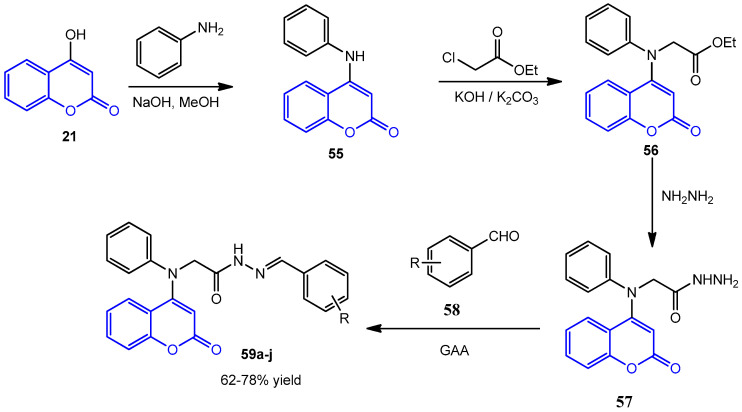
Synthesis of coumarins **59a**–**j** from 4-hydroxy coumarin **21** [80].

Yadav et al. (2022) synthesized 4-anilinocoumarins in four steps (Scheme 13) [80]. At first, compound **55** was prepared via the reaction of aniline with 4-hydroxycoumarin **21**. Next, **55** was treated with ethyl chloroacetate in the presence of KOH/K_2_CO_3_ to give the *N*-alkylated product **56**. The reaction of this intermediate with hydrazine hydrate yielded the desired hydrazide compound **57**, which, upon condensation with aromatic aldehydes **58**, gave 4-anilinocoumarins **59a**–**j** in good yields.

**Scheme 14:**
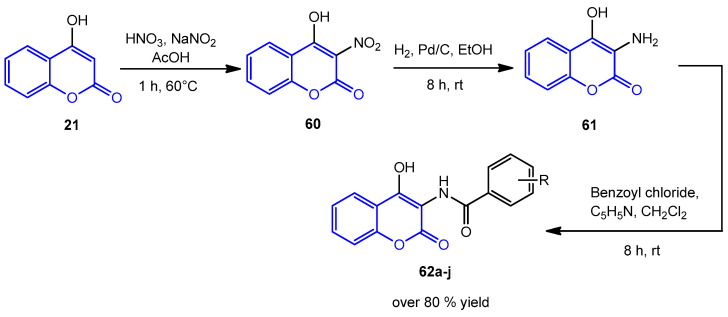
Synthesis of coumarins **62a**–**j** from 4-hydroxy coumarin **21** [81].

With the same purpose of developing coumarin derivatives, Zhou et al. (2022) converted 4-hydroxycoumarin **21** to nitro product **60** (Scheme 14) [81]. The subsequent reduction of the nitro group gave the amino compound **61**, which underwent substitution with various acid chlorides to form the target compounds **62a**–**j** in good yields.

**Scheme 15:**
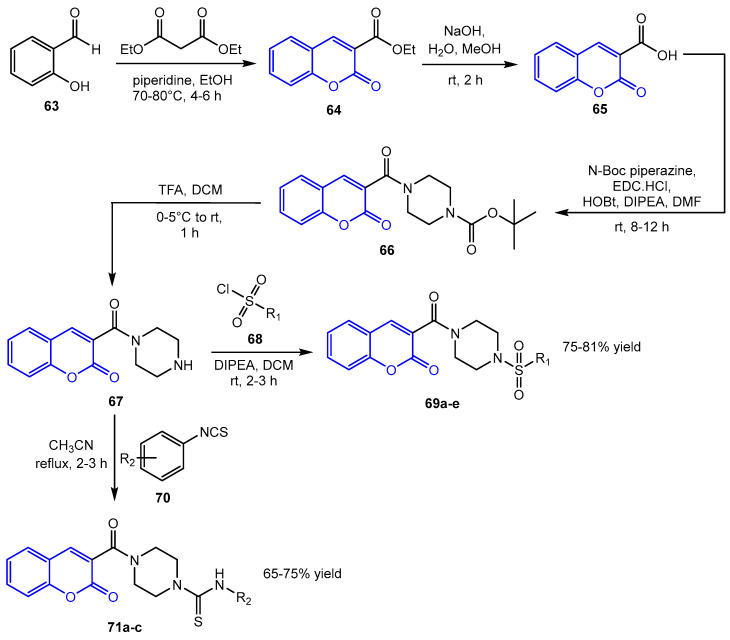
Synthesis of coumarins **69a**–**e** and **71a**–**c** from salicylaldehyde **63** [82].

Ghouse et al. (2023) synthesized 3-substituted coumarin derivatives, starting from salicylaldehyde **63** (Scheme 15) [82]. A reaction between salicylaldehyde **63** and diethyl malonate gave compound **64** which, upon hydrolysis with sodium hydroxide, yielded compound **65**. The amidation of **65** with *N*-Boc piperazine in the presence of EDC·HCl, HOBt, and DIPEA gave **66**. Deprotection of the Boc group using TFA led to the formation of compound **67**. The reaction of **67** with various benzenesulfonylchlorides **68** yielded sulfonamides **69a**–**e** in good yields. On the other hand, **67** underwent a reaction with phenylisothiocyanates **70** to give carbothioamides **71a**–**c** in good yields. 

**Scheme 16:**
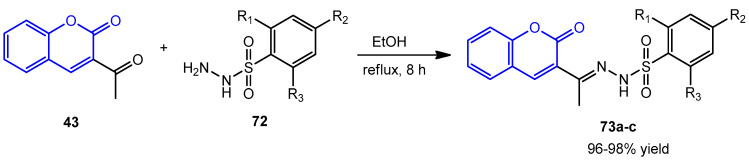
Synthesis of coumarins **73a**–**c** from 3-acetyl coumarin **43** [83].

To obtain coumarin derivatives **73a**–**c**, Prathap and Lokanath (2018) performed a condensation between 3-acetyl coumarin **43** and substituted benzene sulfonyl hydrazides **72** (Scheme 16) [83].

### 2.2. Synthesis of Coumarin–Chalcone Molecular Hybrids

**Scheme 17:**
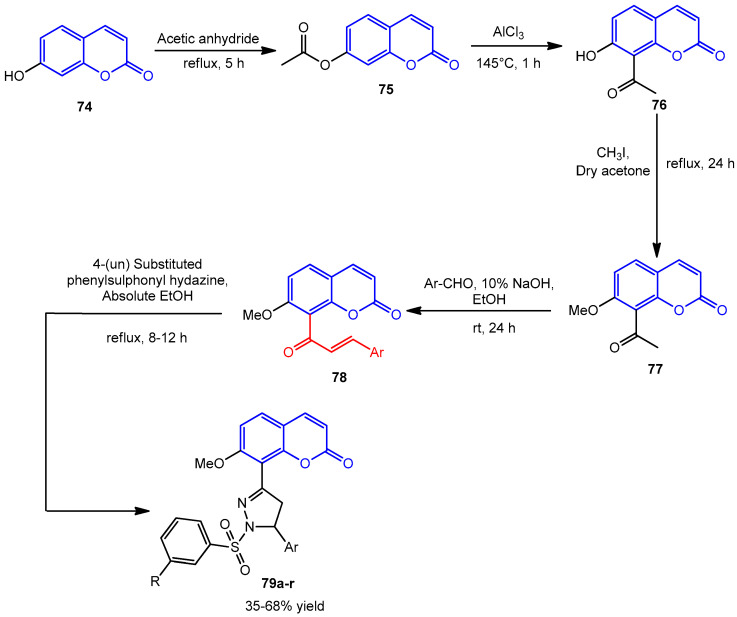
Synthesis of coumarin–chalcones **78** from 7-hydroxy coumarin **74** [84].

Several research groups successfully attempted the synthesis of coumarin–chalcone molecular hybrids using different approaches. The synthesis of the novel coumarin–chalcone hybrid **78** was accomplished by Amin et al. (2013) (Scheme 17) [84]. The synthesis of the precursor chalcone **76** started by converting 7-hydroxycoumarin **74** to acetylated coumarin **75**, followed by acetylation/deacetylation to give **76**. The methylation of **76** gave compound **77**. The aldol condensation of **77** with various aromatic aldehydes gave coumarin–chalcone **78**. These compounds can be further functionalized to **79a**–**r** by reaction of **78** with 4-(un)-substituted phenylsulfonyl hydrazines.

**Scheme 18:**
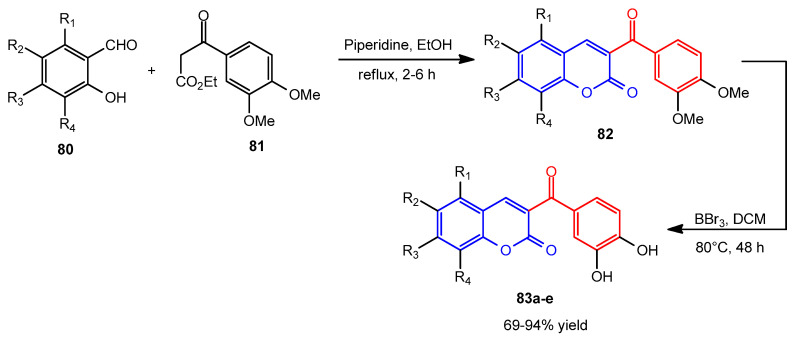
Synthesis of coumarin–chalcones **83a**–**e** from substituted salicylaldehyde **80** [85].

Vazquez-Rodriguez et al. (2013) synthesized hydroxy-3-benzoylcoumarins by initially reacting salicylaldehyde **80** with ethyl 3,4-dimethoxybenzoylacetate **81** to form the Knoevenagel product methoxy-3-benzoylcoumarins **82**, which, upon hydrolysis, gave the target hydroxyl-3-benzoylcoumarins **83a**–**e** in medium-to-good yields (Scheme 18) [85]. 

**Scheme 19:**
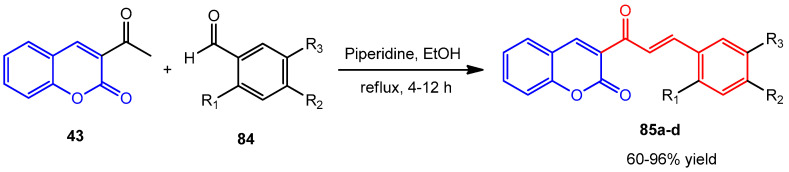
Synthesis of coumarin–chalcones **85a**–**d** from 3-acetyl coumarin **43** [86].

Rodríguez (2015) synthesized coumarin–chalcone derivatives through a two-step process, starting with the preparation of 3-acetyl coumarin **43** via a Knoevenagel reaction. The final coumarins **85a**–**d** were obtained through a Claisen–Schmidt condensation between **43** and aromatic aldehydes **84**, giving good-to-excellent yields (Scheme 19) [86]. 

**Scheme 20:**
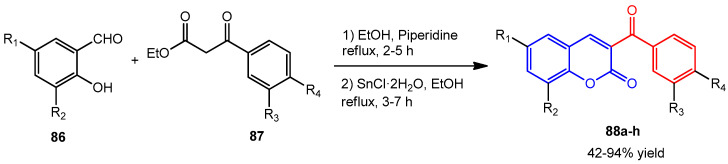
Synthesis of coumarin–chalcones **88a**–**h** from substituted salicylaldehyde **86** [87].

Vazquez-Rodriguez et al. (2015) synthesized coumarin–chalcone derivatives by reacting salicylaldehyde **86** with substituted ethyl benzoylacetate **87** via a Knoevenagel reaction to give derivatives **88a**–**h** (Scheme 20) [87]. In the preparation of amino-substituted derivatives, the same strategy was applied to obtain nitro-substituted 3-benzoyl coumarin precursors, which were further processed without purification using SnCl_2_·2H_2_O. The target compounds **88a**–**h** were obtained with moderate-to-excellent yields. 

**Scheme 21:**
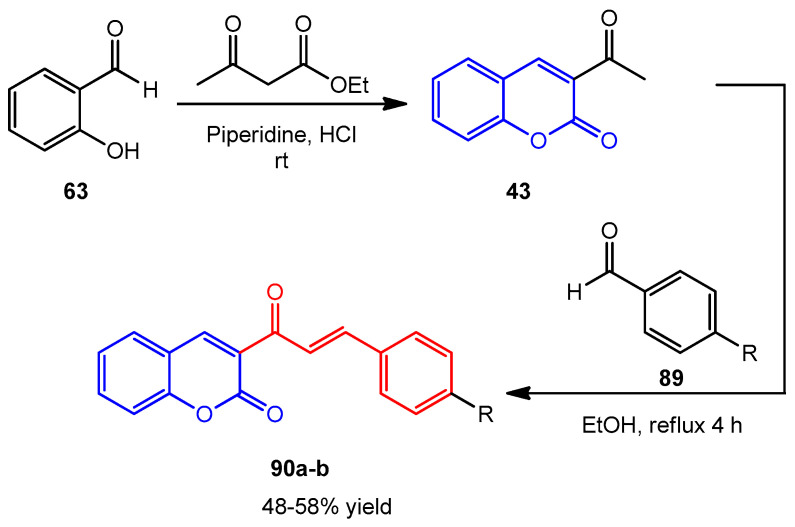
Synthesis of coumarin–chalcones **90a**–**b** from salicylaldehyde **63** [88].

Patil et al. (2019) synthesized coumarin–chalcone derivatives by reacting salicylaldehyde **63** with ethyl acetoacetate to give compound **43** which, upon reaction with aromatic aldehydes **89**, gave the α,β-unsaturated carbonyl compounds **90a**–**b** in moderate yields (Scheme 21) [88]. 

**Scheme 22:**
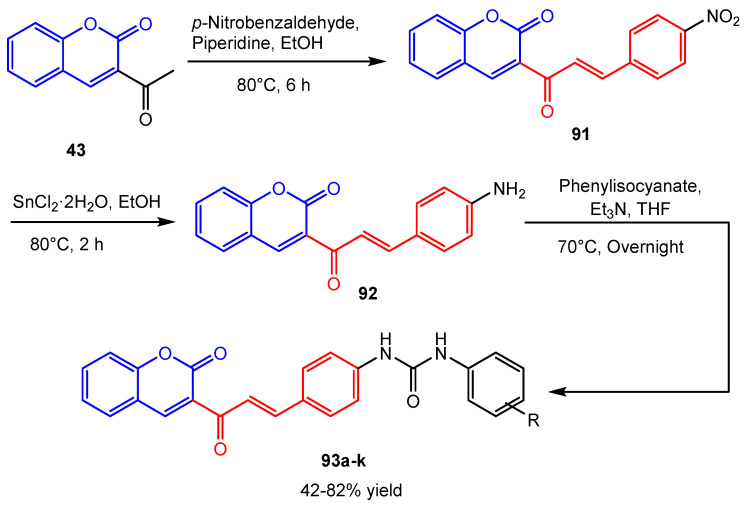
Synthesis of coumarin–chalcones **93a**–**k** from 3-acetyl coumarin **43** [89].

Kurt et al. (2020) synthesized new coumarin–chalcone derivatives (Scheme 22) [89] through Claisen–Schmidt condensation between 3-acetyl coumarin **43** and *p*-nitrobenzaldehyde to give the desired compound **91**, which was then subjected to reduction reaction to give compound **92**. Coumarin–chalcone-substituted urea derivatives **93a**–**k** were obtained in moderate-to-good overall yields by reacting **92** with various isocyanates.

**Scheme 23:**
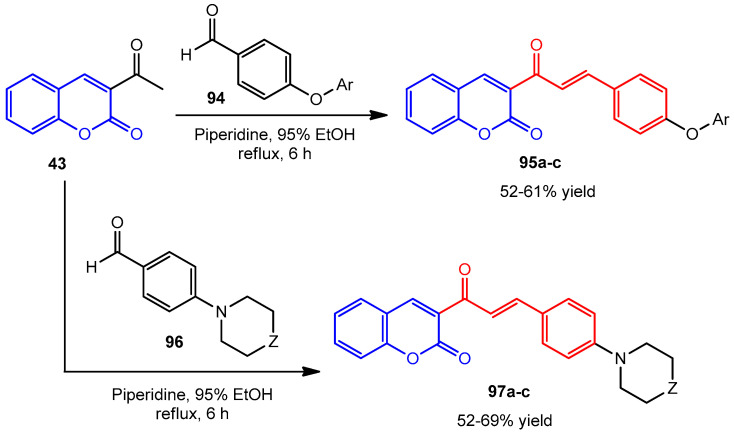
Synthesis of coumarin–chalcones **95a**–**c** and **97a**–**c** from 3-acetyl coumarin **43** [90].

Emam et al. (2021) prepared coumarin–chalcone derivatives via the synthesis route outlined in Scheme 23 [90]. The process involved the Claisen–Schmidt condensation of 3-acetyl coumarin **43** with aryloxybenzaldehydes **94** or dialkylaminobenzaldehydes **96** to give the desired chalcones **95a**–**c** and **97a**–**c**.

**Scheme 24:**
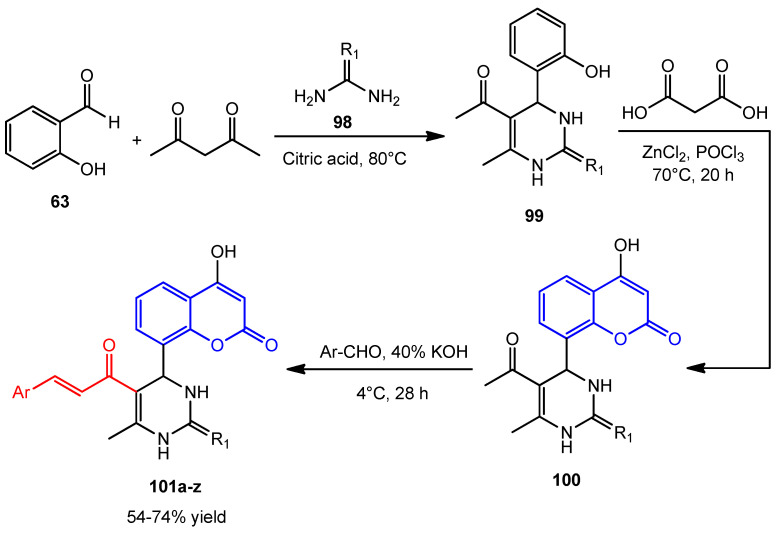
Synthesis of coumarin–chalcones **101a**–**z** from salicylaldehyde **63** [91].

Konidala et al. (2021) synthesized coumarin–clubbed chalcone hybrids as outlined in Scheme 24 [91]. Initially, the acid-catalyzed Biginelli reaction of salicylaldehyde **63** with pentane-2,4-dione and urea/thiourea **98** yielded a dihydropyrimidine derivative **99**. Subsequently, the Pechmann condensation of compound **99** with malonic acid formed compound **100**. Finally, the base-catalyzed Claisen–Schmidt condensation of compound **100** with various aromatic aldehydes gave the target compounds **101a**–**z** in medium-to-good yields.

**Scheme 25:**
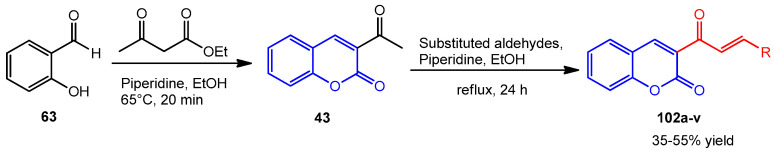
Synthesis of coumarin–chalcones **102a**–**v** from salicylaldehyde **63** [92].

Coumarin–chalcone derivatives were successfully synthesized by Hu et al. (2022) via the reaction of salicylaldehyde **63** with ethyl acetoacetate to give 3-acetyl coumarin **43**; then, subsequent aldol condensation with various aldehydes gave coumarin–chalcone derivatives **102a**–**v** in moderate-to-good yields (Scheme 25) [92].

### 2.3. Synthesis of Coumarin–Triazole Molecular Hybrids

**Scheme 26:**
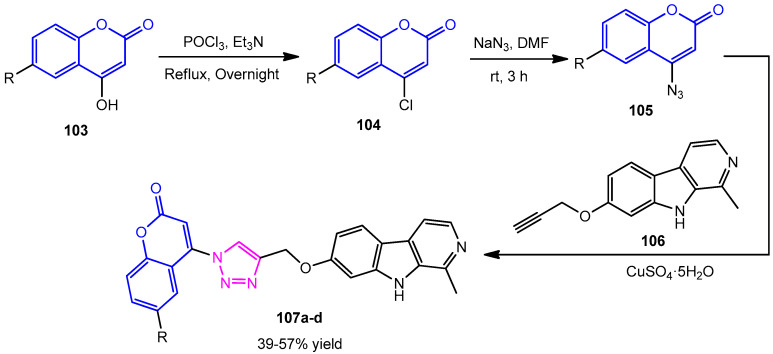
Synthesis of coumarin–triazoles **107a**–**d** from **103** [93].

Coumarin–triazole syntheses were reported from various starting materials. Pavić et al. (2021) synthesized coumarin–triazole hybrids, by initially chlorinating substituted 4-hydroxy coumarin **103** (Scheme 26) [93]**.** The obtained 4-chloro coumarin **104** was then converted to azides **105**. The azide–alkyne cycloaddition was used to produce the target products **107a**–**d** in moderate yields by reacting the azides **105** with the harmine-based terminal alkynes **106**.

**Scheme 27:**
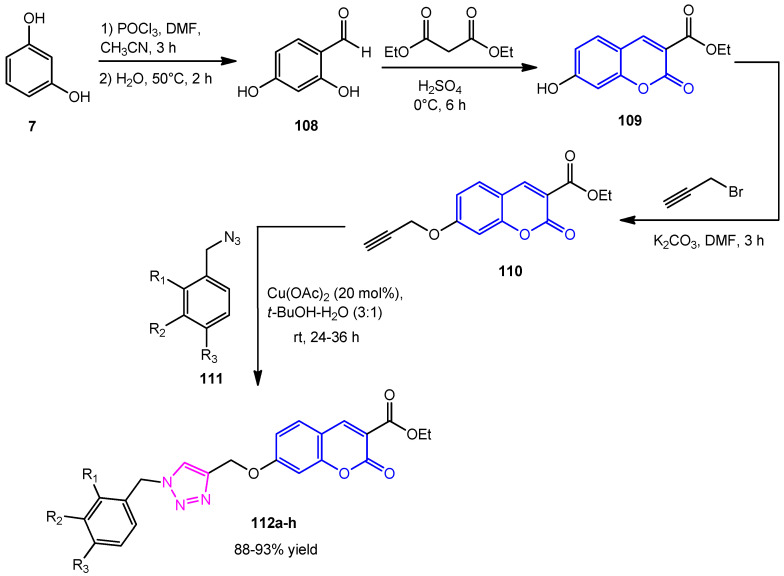
Synthesis of coumarin–triazoles **112a**–**h** from resorcinol **7** [94].

Shaikh et al. (2016) have described the synthesis of a series of new coumarin–triazole derivatives **112a**–**h** from resorcinol **7** (Scheme 27) [94]. Compound **109** was synthesized through the Pechmann condensation between compound **108** and diethyl malonate. The treatment of compound **109** with propargyl bromide resulted in compound **110**. Finally, the reaction of benzyl azide **111** and coumarin-based alkyne **110** via click chemistry produced the corresponding coumarin compounds containing 1,4-disubstituted-1,2,3-triazoles **112a**–**h** in excellent yields.

**Scheme 28:**
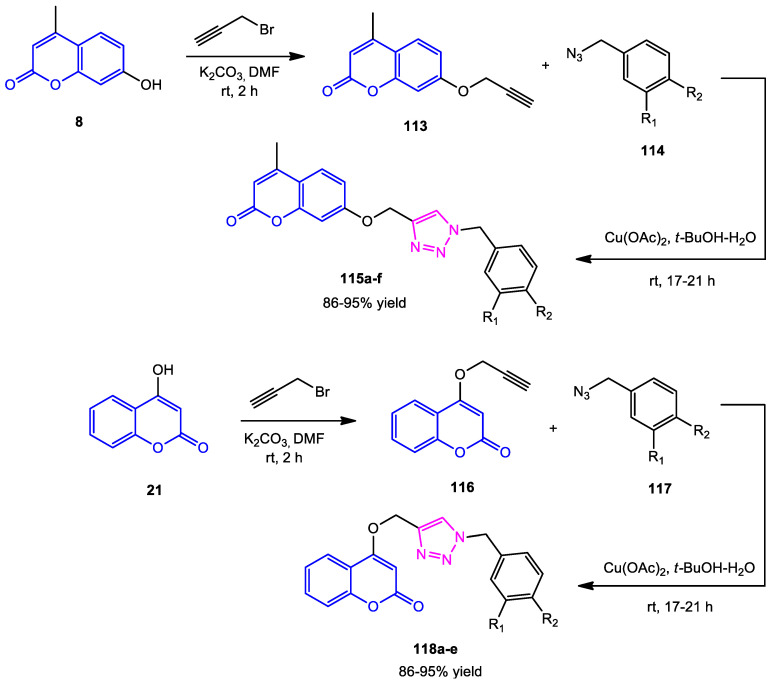
Synthesis of coumarin–triazoles **115a**–**f** and **118a**–**e** from **8** and **21** [95].

Shaikh et al. (2016) described a protocol for the synthesis of a series of coumarin–triazoles **115a**–**f** and **118a**–**e** from benzyl azides and coumarin-based alkynes via click chemistry (Scheme 28) [95]. The reaction between compounds **8** (or **21**) and propargyl bromide produced compounds **113** or **116**, respectively. Then, the reaction between **113** and benzyl azide **114** as well as the reaction of coumarin-based alkynes **116** with **117** gave the corresponding coumarin-based 1,4-disubstituted-1,2,3-triazole derivatives **115a**–**f** or **118a**–**e**, respectively, in excellent yields.

**Scheme 29:**
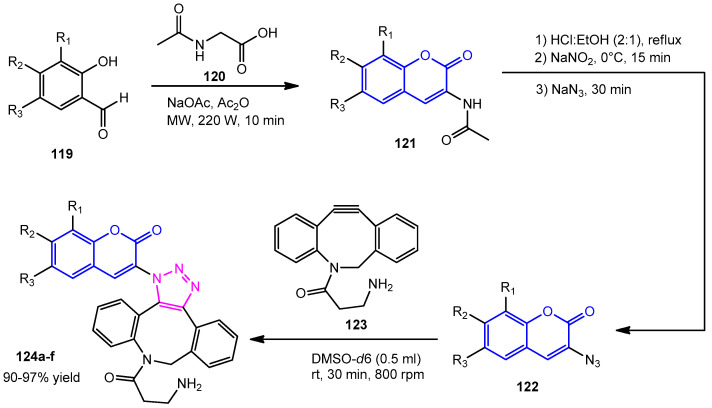
Synthesis of coumarin–triazoles **124a**–**f** from substituted salicylaldehyde **119** [96].

Sinha et al. (2016) developed a new class of coumarin–triazoles through the application of the 1,3-dipolar cycloaddition of azides and alkynes (Scheme 29) [96]. Initially, 3-acetamido coumarin analogs **121** were synthesized using salicylaldehydes **119** and *N*-acetyl glycine **120** under microwave conditions. The subsequent, reaction of compound **121** with sodium nitrite followed by sodium azide gave the desired 3-azido coumarin derivatives **122**. Next, a click reaction between cyclooctyne **123** and 3-azido coumarins **122** gave compounds **124a**–**f** in excellent yields.

**Scheme 30:**
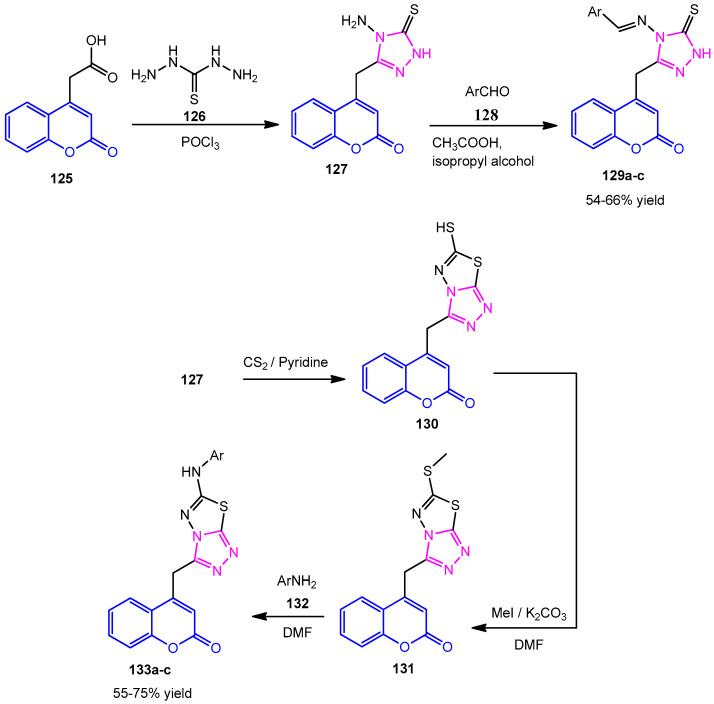
Synthesis of coumarin–triazoles **129a**–**c** and **133a**–**c** from **125** [97].

The synthesis of the target triazolyl coumarin derivatives by Al-Wahaibi et al. (2018) utilized 2-(coumarin-4-yl) acetic acid **125**, which was prepared by reacting citric acid with phenol in sulfuric acid (Scheme 30) [97]. Compound **125** was then reacted with thiocarbohydrazide **126** to produce compound **127**. The reaction of aminotriazole **127** with various aromatic aldehydes **128** using acetic acid as a catalyst gave the arylideneamino derivatives **129a**–**c** in medium yields. The reaction of compound **127** with carbon disulfide in pyridine gave the mercapto derivative **130**. The mercapto derivative **130** was then reacted with iodomethane to produce the methylthio analogue **131**. The reaction of **131** with various primary aromatic amines **132** gave the desired 6-arylamino compounds **133a**–**c** in good yields.

**Scheme 31:**
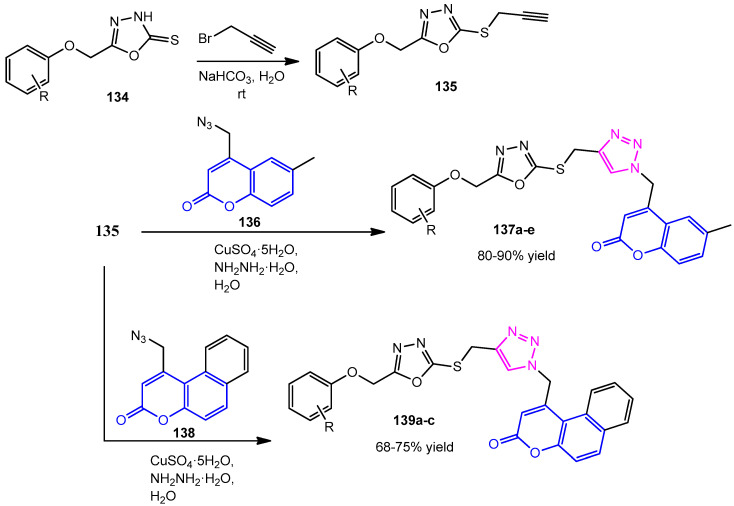
Synthesis of coumarin–triazoles **137a**–**e** and **139a**–**c** from **134** [98].

Kumar et al. (2018) successfully synthesized coumarin derivatives containing a 1,2,3-triazole ring (Scheme 31) [98]. The chemistry began by reacting 5-substituted-2-mercapto-1,3,4-oxadiazole **134** with propargyl bromide to give the compound **135**, which was used for the click chemistry. Compound **135** underwent a 1,3-dipolar cycloaddition with 4-azido methyl coumarins **136** or **138** to yield compounds **137a**–**e** and **139a**–**c**, respectively, in good yields.

**Scheme 32:**
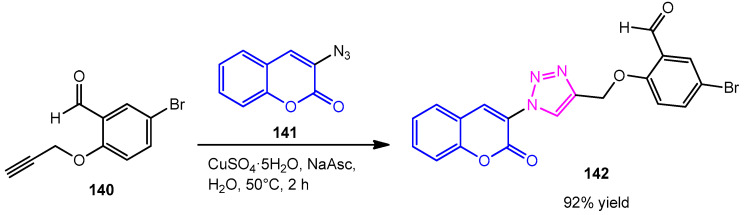
Synthesis of coumarin–triazole **142** [99].

Nouraie et al. (2019) successfully synthesized coumarin-1,2,3-triazole hybrids in excellent yields (Scheme 32) [99]. For example, the reaction of 5-bromo-2-(prop-2-yn-1-yloxy)benzaldehyde **140** and 3-azido coumarin **141** gave compound **142** in a 92% yield.

**Scheme 33:**
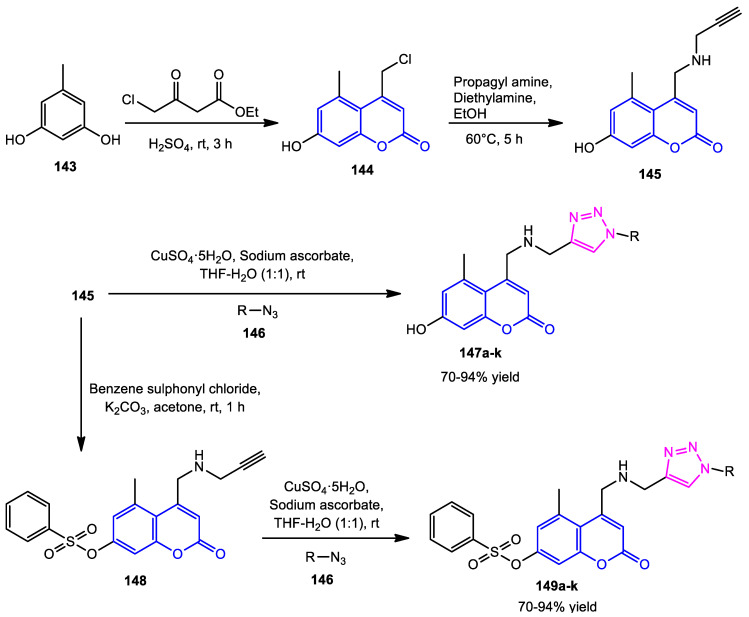
Synthesis of coumarin–triazoles **147a**–**k** and **149a**–**k** from orcinol **143** [100].

Coumarin-1,2,3-triazole hybrids bearing a benzenesulfonate moiety have been successfully synthesized by Krishna et al. (2018) (Scheme 33) [100]. The Pechmann cyclization of orcinol **143** with ethyl 4-chloroacetoacetate gave compound **144**. Compound **144** was then reacted with propargyl amine, resulting in *N*-propargyl coumarin **145**. The subsequent treatment of coumarin **145** with benzene sulfonyl chloride produced the benzene sulfonate coumarin **148**. The 1,3-dipolar cycloaddition of the resultant acetylenic compounds **145** and **148** with substituted aromatic azides under click chemistry yielded compounds **147a**–**k** and **149a**–**k**, respectively, in good-to-excellent yields.

**Scheme 34:**
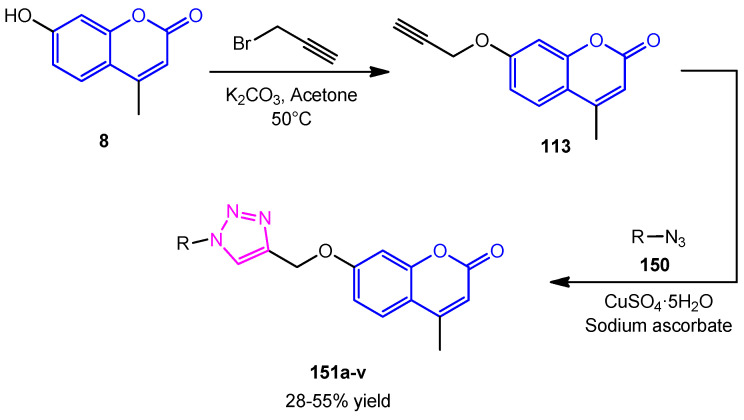
Synthesis of coumarin–triazoles **151a**–**v** from 7-hydroxy-4-methyl coumarin **8** [101].

Yadav et al. (2018) synthesized a series of novel coumarin–triazoles **151a**–**v** by employing click chemistry on 7-hydroxy-4-methyl coumarin **8** (Scheme 34) [101]. Initially, compound **8** was alkylated using propargyl bromide to produce coumarin alkynes **113**. The alkynes **113** underwent a cycloaddition reaction with azides **150** to yield a series of 1,4-substituted triazoles **151a**–**v** in moderate yields.

**Scheme 35:**
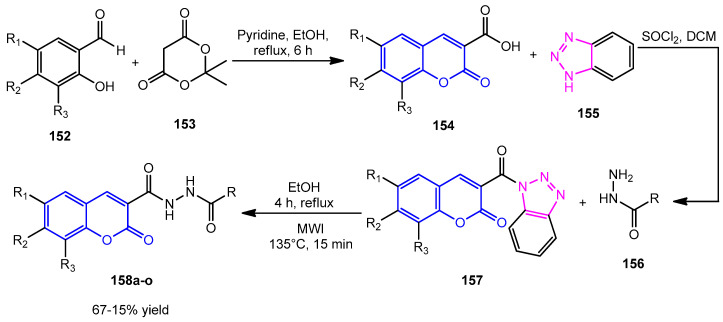
Synthesis of coumarin–triazoles **157** from salicylic aldehydes **152** [102].

Yilmaz and Faiz (2018) successfully synthesized 3-(1*H*-benzotriazol-1-ylcarbonyl)-2*H*-chromen-2-ones **157** from salicyl aldehydes **152** (Scheme 35) [102]. Coumarin-3-carboxylic acids **154** were obtained from the reaction of salicyl aldehydes **152** and Meldrum’s acid **153** with pyridine as a catalyst. Then, compounds **154** were reacted with 1-*H*-benzotriazole **155** to afford compounds **157**. Coumarin triazole **157** can also be further functionalized to **158a**–**o** by reaction with hydrazide **156**. 

**Scheme 36:**
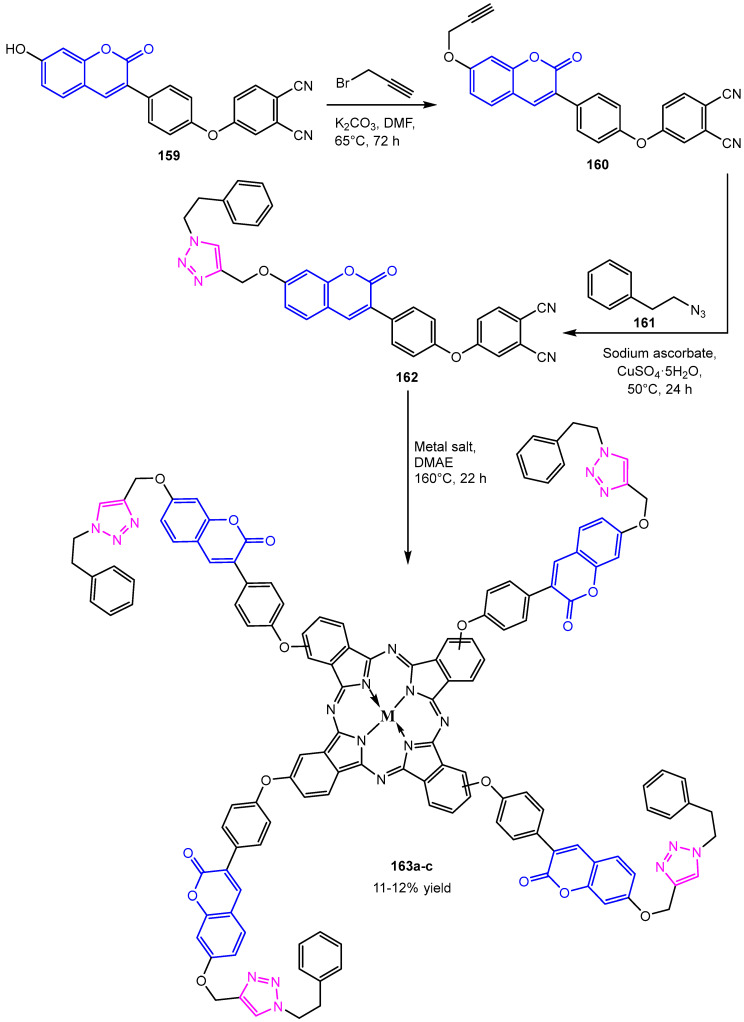
Synthesis of coumarin–triazoles **163a**–**c** from compound **159** [103].

The synthesis of 1,2,3-triazole-containing coumarin bearing Zn(II) and Mg(II) phthalocyanines was successfully carried out by Özdemir et al. (2020) (Scheme 36) [103]. The synthesis began by reacting 7-hydroxy-3-(3,4-dicyanophenoxyphenyl)coumarin **159** with propargyl bromide to produce coumarin alkynes **160**. The 1,2,3-triazoles **162** were synthesized through the copper(I)-catalyzed Huisgen 1,3-dipolar cycloaddition reaction of 2-(azidoethyl)benzene **160** with terminal ethynyl-bearing phthalonitriles **161**. The metallophthalocyanines **163a**–**c** were obtained through the cyclotetramerization of triazole-coumarin phthalonitriles **162** in the presence of metal salts in 2-dimethylaminoethanol (DMAE) as a solvent.

**Scheme 37:**
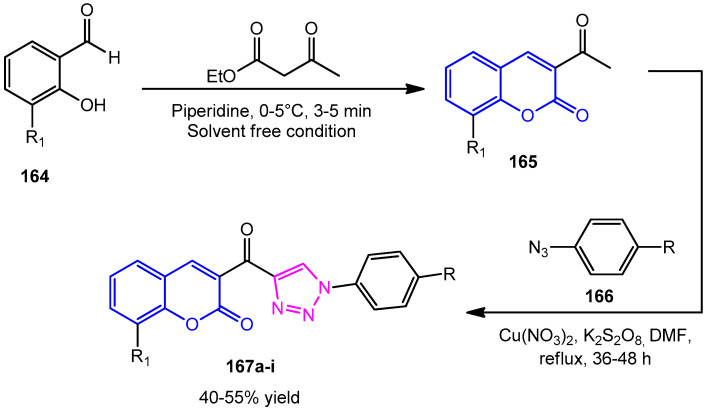
Synthesis of coumarin–triazoles **167a**–**i** from substituted salicylaldehyde **164** [104].

The synthesis of coumarin-tethered 1,2,3-triazoles by Channabasappa et al. (2021) involved a multi-step route (Scheme 37) [104]. Compound **165** was prepared from substituted salicylaldehyde **164** and ethyl 3-oxobutanoate. The aryl azides **166** were reacted with substituted compounds **165**, yielding compounds **167a**–**i** in moderate yields. 

**Scheme 38:**
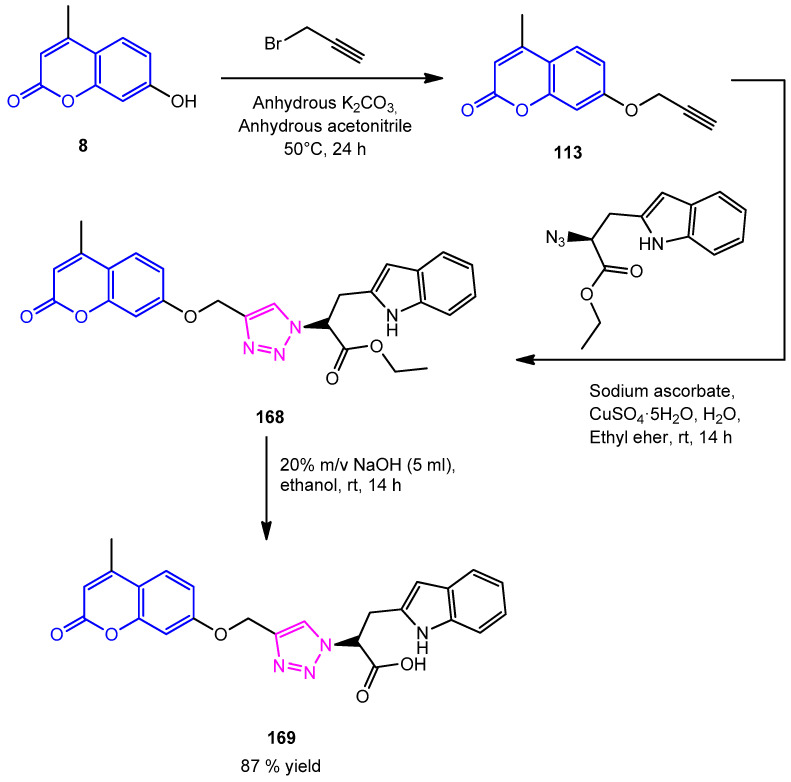
Synthesis of coumarin–triazoles **169** from 7-hydroxy-4-methyl coumarin **8** [105].

The synthesis of coumarin-linked amino acids via triazole was accomplished by De Sousa et al. (2021) (Scheme 38) [105]. Compound **8** was propargylated to afford **113**. Reaction of **113** with azido ethyl ester of tryptophan via azide-alkyne cycloaddition gave coumarin triazole **168**, which was hydrolyzed to yield coumarin-linked triazole amino acid analog **169**. 

**Scheme 39:**
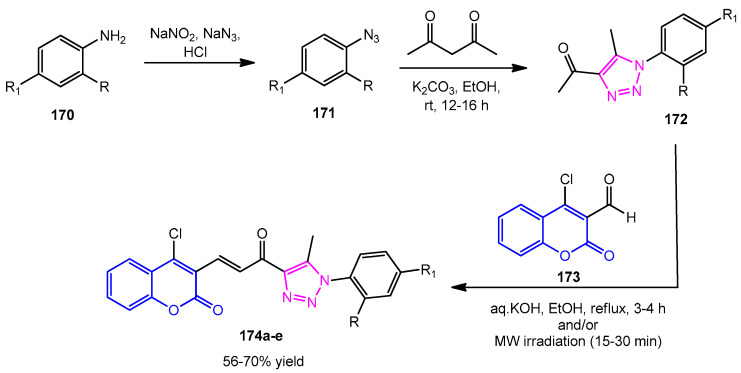
Synthesis of coumarin–triazoles **174a**–**e** from aromatic anilines **170** [106].

A series of new coumarin-1,2,3-triazole hybrids were also synthesized by Vagish et al. (2021) (Scheme 39) [106]. Initially, aniline **170** underwent diazotization followed by azidation to give azido **171**. Key intermediate **172** was prepared via enolate-mediated triazole synthesis from **171** using acetylacetone. Finally, the target coumarin 1,2,3-triazole conjugates **174a**–**e** were synthesized by the reaction of **172** with compound **173** under different conditions.

**Scheme 40:**
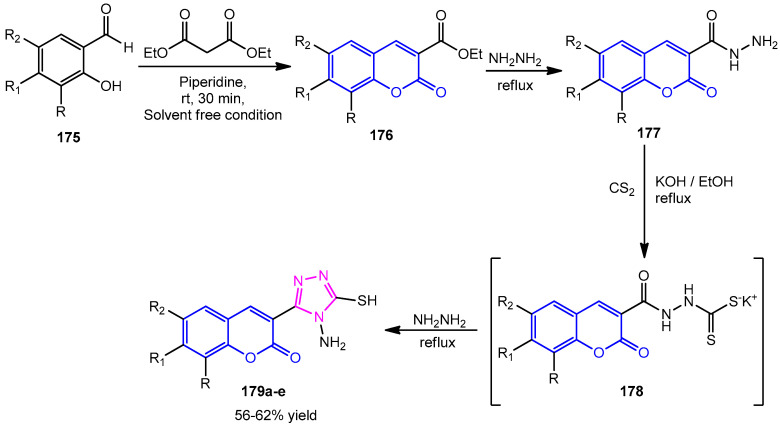
Synthesis of coumarin–triazole **179a**–**e** from substituted salicylaldehyde **175** [107].

In a multi-step route, a series of coumarin–triazole hybrids were successfully synthesized by Basappa et al. (2020) (Scheme 40) [107]. The reaction between substituted salicylaldehydes **175** and diethylmalonate gave compound **176** which, upon reaction with hydrazine hydrate, gave hydrazides **177**. The reaction of **177** with carbon disulfide resulted in the formation of the intermediate dithiocarbazate salts **178**. The in-situ reaction of **178** with hydrazine hydrate produced the target compounds **179a**–**e** in medium-to-good yields.

**Scheme 41:**
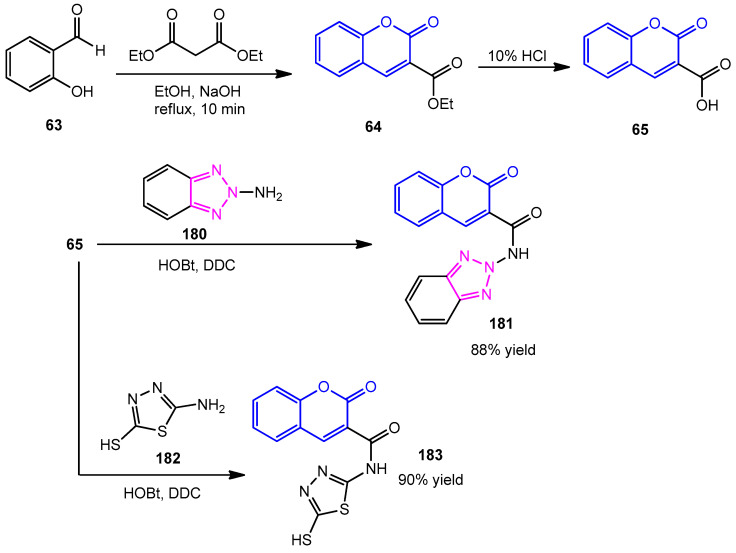
Synthesis of coumarin–triazoles **181**/**183** from salicylaldehyde **63** [108].

The synthesis of new coumarin derivatives containing aminobenzotriazole moiety was successfully carried out by Al-Shuaeeb (2023) (Scheme 41) [108]. A reaction between salicylaldehyde **63** and diethyl malonate gave compound **64** which, upon hydrolysis, yielded coumarin 3-carboxylic acid **65**. Coupling using dicyclohexylcarbodiimide (DCC) was employed to connect coumarin 3-carboxylic acid **65** with 2-aminobenzotriazole **180**, resulting in the formation of compounds **181** in good yield. Moreover, reaction of compound **65** with 2-amino-5-mercapto-1,3,4-thiadiazole **182** gave derivative **183** in good yield.

**Scheme 42:**
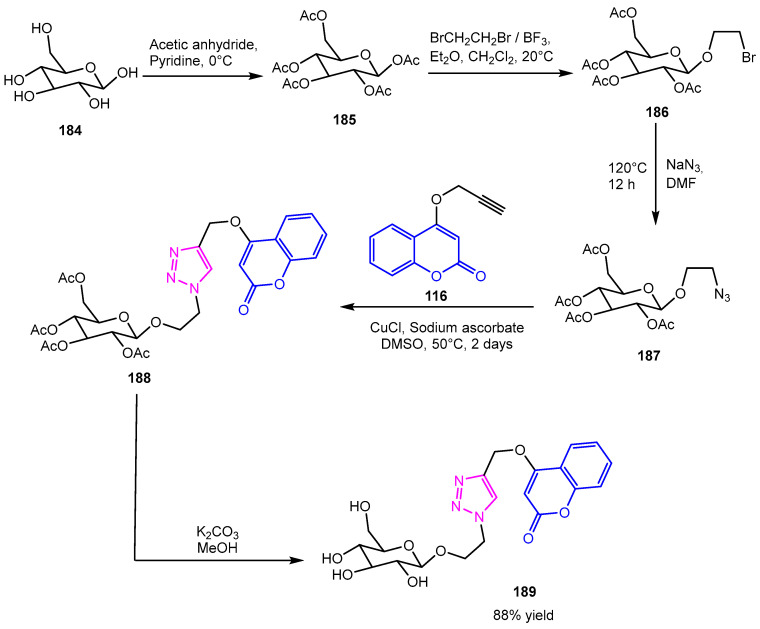
Synthesis of coumarin–triazole **189** from β-d-glucopyranoside **184** [109].

Adam and Zimam (2023) successfully synthesized new coumarin-1,2,3–triazole glucoside hybrids (Scheme 42) [109]. β-D-glucopyranoside **184** was reacted with acetic anhydride in the presence of pyridine to produce compound **185**, which was then reacted with 1,2-dibromoethane to yield compound **186**. Subsequently, compound **186** was reacted with sodium azide to produce azidosugar **187**. The reaction of compound **187** with propargyl 4-hydroxy coumarin **116** occurred under click chemistry conditions to create 1,2,3–triazole glycoside **188**. The acetylated glycoside **188** underwent a deprotection reaction to afford triazole glycoside **189** with free hydroxyl groups.

**Scheme 43:**
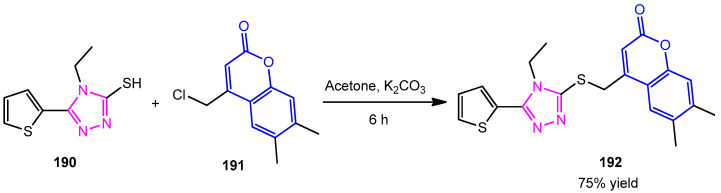
Synthesis of coumarin–triazole **192** from **191** [110].

In 2023, Omar et al. synthesized a novel coumarin–triazole hybrid by reacting 4-ethyl-5-(thiophen-2-yl)-4*H*-1,2,4-triazole-3-thiol **190** with an equimolar amount of 4-(chloromethyl)-6,7-dimethyl-2*H*-chromen-2-one **191** to give coumarin–triazole **192** in 75% yield (Scheme 43) [110].

**Scheme 44:**
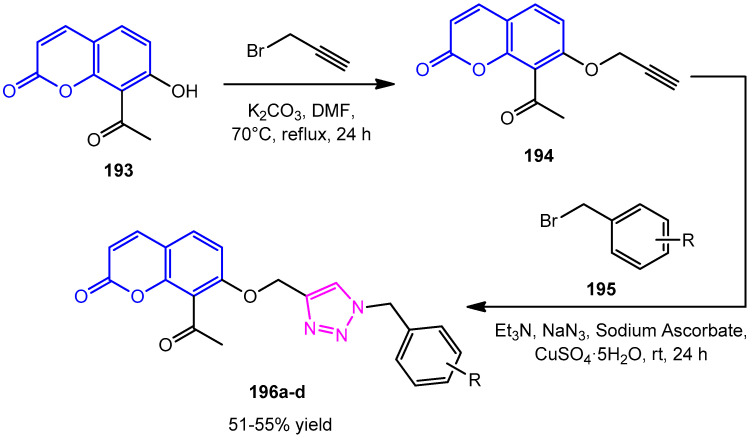
Synthesis of coumarin–triazoles **196a**–**d** from 8-acetyl-coumarin **193** [111].

A series of coumarin−triazole hybrid syntheses conducted by Sharma and Bharate (2023) relied on the propargylation of 8-acetyl-coumarin **193** with propargyl bromide to give compound **194** (Scheme 44) [111]. The click reaction of the propargyl **194** with benzyl bromide **195** in the presence of sodium azide yielded the corresponding coumarin−triazole hybrids **196a**–**d** in medium yields.

**Scheme 45:**
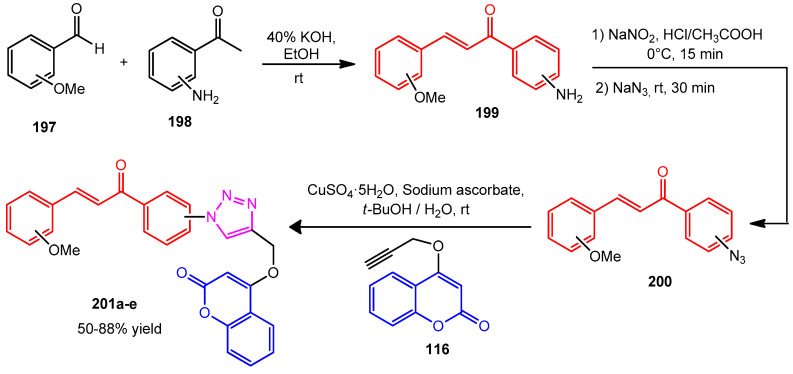
Synthesis of coumarin–triazole–chalcones **201a**–**e** from aromatic aldehydes and ketones [112].

Pingaew et al. (2014) synthesized novel coumarin–triazole–chalcone hybrids in three steps (Scheme 45) [112]. Initially, chalcones **199** were synthesized using the Claisen–Schmidt condensation of various aromatic aldehydes **197** with amino acetophenones **198**. Subsequently, the diazotation–azidation of the aminochalcones **199** afforded the corresponding azidochalcones **200**. The azide–alkyne dipolar cycloaddition is the key route to the synthesis of 1,2,3-triazole linkage. Finally, the azides **200** readily underwent cycloaddition with alkynes **116** to afford the novel desired hybrid molecules **201a**–**e** in medium-to-good yields.

## 3. Bioactivity of Coumarins, Coumarin–Chalcones, and Coumarin–Triazoles

### 3.1. Antioxidant Activity

**Figure 3:**
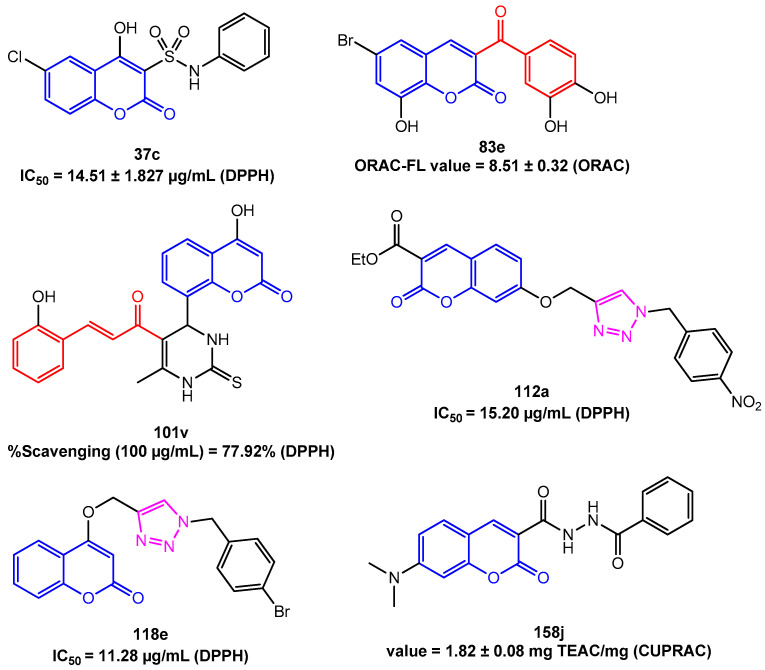
Coumarin-based compounds as antioxidants.

Alshibl et al. (2020) synthesized coumarin derivates and examined their antioxidation activity using a DPPH assay (Scheme 8, Figure 3) [75]. Compound **37c** exhibited the highest antioxidant activity, with an IC_50_ value of 14.51 ± 1.827 μg/mL.

The capacity of hydroxy-3-benzoylcoumarins to scavenge peroxyl radicals was studied using the ORAC method [85]. ORAC assesses the ability of antioxidants (or their complex mixtures) to inhibit the bleaching of a target molecule (probe) induced by peroxyl radicals. The results from the ORAC method showed that compound **83e** (Scheme 18, Figure 3) had the best values among the synthesized compounds. The ORAC-FL value was 8.51 ± 0.32, the ORAC-PGR value was 1.17 ± 0.10, and the hydroxy radical scavenging value was 90.9 ± 8.2% for compound **83e**.

The method of screening the antioxidant activity of coumarin–clubbed chalcone hybrids developed by Konidala et al. (2021) was examined using an in vitro DPPH assay (Scheme 24, Figure 3) [91]. The results indicated that compound **101v** showed the most potent antioxidant activity, with 77.92% scavenging activity (100 μg/mL).

The coumarin–triazole derivatives developed by Shaikh et al. were also evaluated for antioxidant activity using in vitro DPPH assay. Compound **112a** (Scheme 27, Figure 3) was found to be the most potent candidate, with an IC_50_ value of 15.20 μg/mL [94].

Coumarin–triazole **118e** (Scheme 28, Figure 3), assessed using an in vitro DPPH radical scavenging assay, showed an IC_50_ of 11.28 μg/mL [95].

The potential antioxidant activity of several coumarin hydrazides was also investigated using the cupric ion reducing antioxidant capacity (CUPRAC) method. All studied compounds reduced cupric ions, and the highest activity was observed for compound **158j**, with 1.82 ± 0.08 mg TEAC/mg compound (Scheme 35, Figure 3) [102].

### 3.2. Antimicrobial Activity

**Figure 4:**
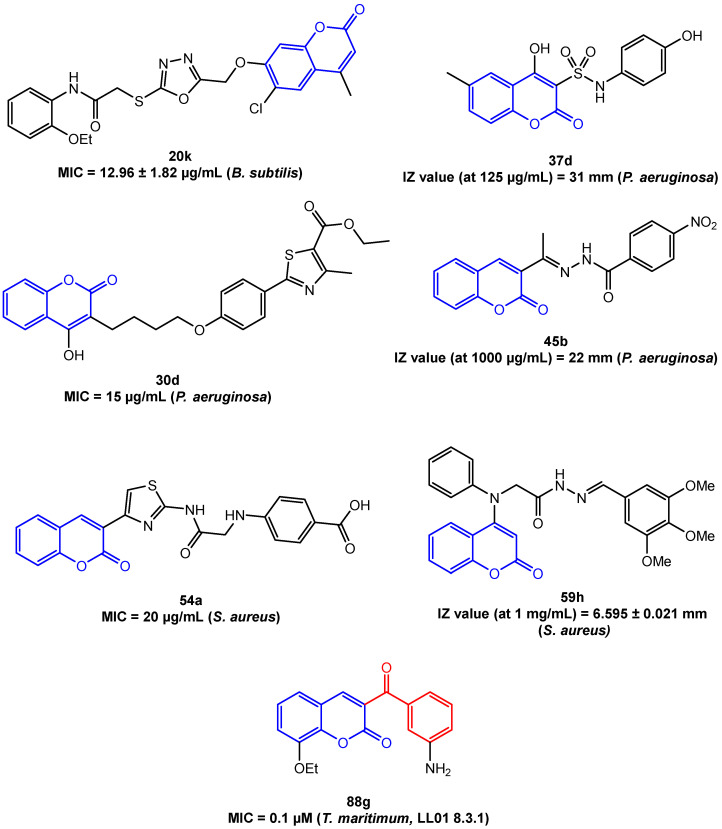
Coumarin-based compounds as antimicrobial agents.

Rasool et al. (2016) reported several coumarin hybrids as potential antimicrobial agents. Compound **20k** (Scheme 3, Figure 4) was found to be the most potent candidate, with promising MIC values for Gram-negative bacteria (*S. typhi* = 8.84 ± 2.00 μg/mL; *E. coli* = 10.94 ± 1.25 μg/mL; *P. aeruginosa* = 12.00 ± 1.33 μg/mL) and Gram-positive bacteria (*B. subtilis* = 12.96 ± 1.82 μg/mL; *S. aureus* = 12.14 ± 1.48 μg/mL) [70].

Naik et al. (2019) synthesized novel coumarin derivatives, and the best antimicrobial activity was shown by compound **30d** (Scheme 6, Figure 4), with promising MIC values against Gram-positive bacteria (*E. coli* = 14 μg/mL; *P. aeruginosa* = 15 μg/mL) and Gram-negative bacteria (*B. subtilis* = 14 μg/mL; *S. aureus* = 10 μg/mL) [73].

Additionally, Alshibl et al. (2020) successfully synthesized and tested coumarin derivates and found compound **37d** (Scheme 8, Figure 4) to possess the highest IZ at a concentration of 125 μg/mL. The antimicrobial activity, expressed as the diameter (mm) of inhibition zones, for Gram-positive bacteria was *S. aureus* = 30 mm, *B. subtilis* = 29 mm, and *B. megaterium* = 29 mm; for Gram-negative bacteria, the antimicrobial activity was *E. coli* = 31 mm and *P. aeruginosa =* 31 mm, and for fungi it was *S. cerevisiae* = 32 mm and *C. albicans* = 30 mm [75]. 

Abduljabbar and Hadi (2021) constructed several coumarin derivatives and tested their antimicrobial activity using the disc-well-diffusion method [77]. The highest activity was demonstrated by compound **45b** (Scheme 10, Figure 4), which showed the highest IZs at a concentration of 1000 μg/mL (*P. aeruginosa* = 22 mm and *E. coli* = 14 mm).

Other coumarin derivatives were assessed in vitro using the cup plate method for the MIC [79]. Compound **54a** (Scheme 12, Figure 4) exhibited the best activity, with promising MIC values for Gram-negative bacteria (*E. coli* = 40 μg/mL; *P. aeruginosa* = 30 μg/mL), for Gram-positive bacteria (*B. subtilis* = 30 μg/mL; *S. aureus* = 20 μg/mL), and for fungi (*A. niger* = 30 μg/mL; *C. albicans* = 40 μg/mL).

The antimicrobial activity of novel 4-anilinocoumarin derivatives was also investigated using the zone inhibition assay/well-diffusion assay [80]. Compound **59h** (Scheme 13, Figure 4) showed good activity for Gram-negative bacteria (*E. coli* = 3.755 ± 0.091 mm; *P. aeruginosa* = 5.66 ± 0.014 mm) and Gram-positive bacteria (*B. subtilis* = 5.335 ± 0.021 mm; *S. aureus* = 6.595 ± 0.021 mm).

The antimicrobial activity screening of coumarin–chalcone hybrids developed by Vazquez-Rodriguez et al. (2015) was examined in vitro for *T. maritimum* strains [87]. All *T. marirtimum* strains were highly sensitive to compound **88g** (Scheme 20, Figure 4). The MIC and MBC were evaluated in eight of the *T. maritimum* with the following results: NCIMB 2154—MIC = 62.5 μM, MBC = 62.5 μM; Tm Chile—MIC = 62.5 μM, MBC = 62.5 μM; LP2R—MIC = 62.5 μM, MBC = 125 μM; JIP 31/99—MIC = 15.6 μM, MBC = 15.6 μM; LL01 8.3.8.—MIC = 0.1 μM, MBC = 0.1 μM; LL01 8.3.1—MIC = 0.1 μM, MBC = 0.1 μM; Dba4a—MIC = 0.5 μM, MBC = 1.9 μM; and 3.35—MIC = 1.0 μM, MBC = 1.9 μM.

**Figure 5:**
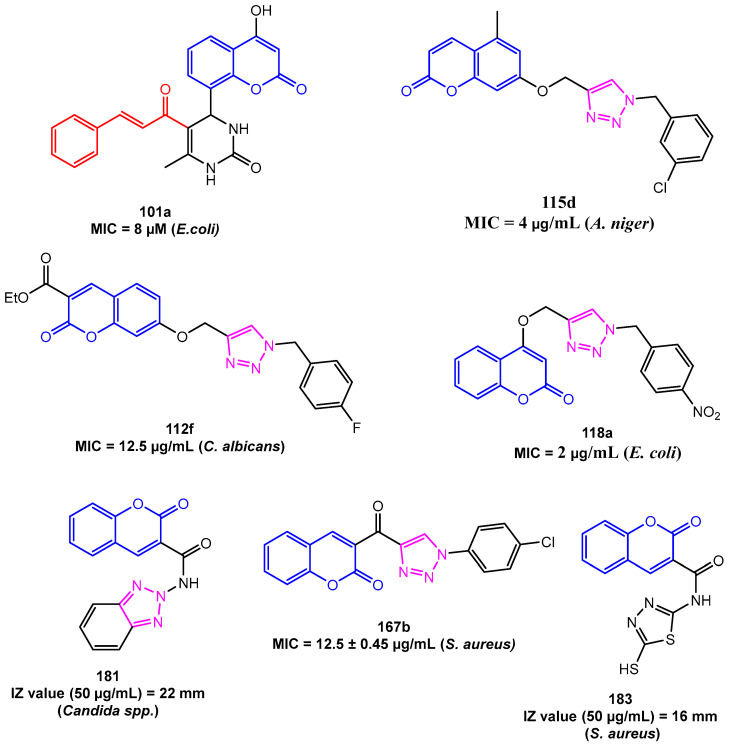
More examples of coumarin-based compounds as antimicrobial agents.

The coumarin–clubbed chalcone hybrids developed by Konidala et al. (2021) [91] were also evaluated in vitro using the well-diffusion method. Compound **101a** (Scheme 24, Figure 5) was the most potent, with an MIC value of 10 μM against a Gram-positive bacterium (*Staphylococcus aureus*), an MIC value of 8 μM against a Gram-negative bacterium (*Escherichia coli*), and an antifungal activity MIC value of 10 μM against *Aspergillus niger*.

Shaikh et al. (2016) synthesized a series of new coumarin–triazole derivatives and tested their antimicrobial activity [94]. Compound **112f** (Scheme 27, Figure 5) displayed significant antifungal activity (in comparison to the standard antifungal drug miconazole), with the following MIC values: *Candida albicans* = 12.5 μg/mL, *Fusarium oxysporum* = 50 μg/mL, *Aspergillus flavus* = 50 μg/mL, *Aspergillus niger* = 25 μg/mL, and *Cryptococcus neoformans* = 100 μg/mL.

The antimicrobial activity of a series of novel coumarin–triazole hybrids was also investigated. Compound **115d** (Scheme 28, Figure 5) exhibited the best activity, with the following MIC values for fungi: *Aspergillus Niger* = 4 μg/mL, *Penicillium Chrysogenum* = 4 μg/mL, and *Curvularia Lunata* = 8 μg/mL. When compound **115d** was docked into the active site of the cytochrome P450 lanosterol 14α-demethylase of *C. albicans* using VLifeMDS 4.3 software, it showed the lowest interaction energy of −72.29 kcal/mol [95].

Shaikh et al. (2016) also successfully synthesized and tested the antimicrobial activity of coumarin–triazole derivates [95]. Compound **118a** (Scheme 28, Figure 5) showed excellent inhibitory activity, with MIC values of 8 μg/mL, 4 μg/mL, 4 μg/mL, 2 μg/mL, 2 μg/mL, and 2 μg/mL against *Staphylococcus aureus*, *Micrococcus luteus*, *Bacillus cereus*, *Escherichia coli*, *Pseudomonas fluorescens*, and *Flavobacterium devorans*, respectively.

1,2,3-Triazole **167b** (Scheme 37, Figure 5) showed potent inhibition, with MIC values against *S. aureus* (12.5 ± 0.45 μg/mL), *E. coli* (25.0 ± 1.20 μg/mL), *P. aeruginosa* (12.5 ± 0.90 μg/mL), *A. niger* (25.0 ± 0.65 μg/mL), *A. flavus* (25. ± 0.65 μg/mL), and *C. albicans* (12.5 ± 0.60 μg/mL) [104].

The antimicrobial activity of coumarin derivatives containing aminobenzotriazole and triazole moieties indicated compounds **181** and **183** (Scheme 41, Figure 5), with the highest IZs at a concentration of 50 μg/mL. The antimicrobial activity of compounds **181** and **183**, expressed as the diameter (mm) of the inhibition zone, is as follows: Gram-positive (*Staphylococcus aureus* = 15 and 16 mm; *Streptococcus aureus* = 12 and 14 mm), Gram-negative (*Proteus* spp. = 7 and 9 mm), and fungi (*Aspergillus* spp. = 19 and 9 mm; *Candida* spp. = 22 and 7 mm) [108].

### 3.3. Anticancer Activity

**Figure 6:**
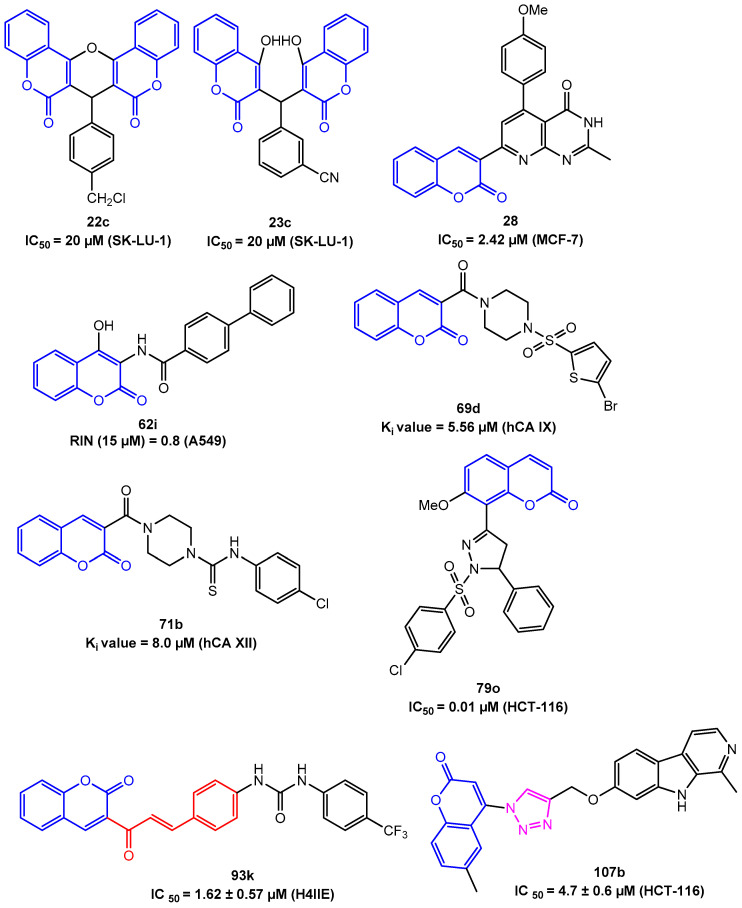
Coumarin-based compounds as anticancer agents.

Weng and Yuan (2017) successfully synthesized and tested two types of coumarin derivatives against a human lung cancer cell line [71]. Among the investigated compounds, compounds **22c** and **23c** (Scheme 4, Figure 6) exhibited the most potent growth inhibition, with compound **22c** showing IC_50_ values of SK-LU-1 = 20 μM, SPC-A-1 = 20 μM, and 95D = 23 μM and compound **23c** showing IC_50_ values of SK-LU-1 = 20 μM, SPC-A-1 = 21 μM, and 95D = 25 μM.

Fayed et al. (2019) examined the anticancer activity of several coumarin derivatives using an MTT assay. Compound **27** (Scheme 5, Figure 6) displayed significant anticancer activity, with IC_50_ values of MCF-7 = 2.42 μM, HCT-116 = 6.11 μM, HepG-2 = 13.32 μM, and A549 = 17.21 μM. Docking studies showed that compound **27** fits in the binding pocket of caspase-3, and the binding energy was −6.224 kcal/mol [72].

The coumarin derivatives tested by Zhou et al. (2022) [81] showed compound **62i** (Scheme 14, Figure 6) to exhibit the best in vitro activity against lung cancer cell motility via an analysis of invasion assay cells treated with 15 μM of the compound. The values of the Relative Invaded Number (RIN) for each lung cancer cell are as follows: A549 = 0.8, H460 = 0.8, H1650 = 0.75, and H1975 = 0.78.

Ghouse et al. (2023) synthesized 3-substituted coumarin derivatives and examined their anticancer activity using their CA inhibitory potential against the isoforms hCA I, II, IX, and XII [82]. The results showed that compounds **69d** and **71b** (Scheme 15, Figure 6) exhibited the best K_i_ values. Compound **69d** showed hCA I, K_i_ > 100 μM; hCA II, K_i_ > 100 μM; hCA IX, K_i_ = 5.56 μM; hCA XII, K_i_ = 9.8 μM, and compound **71b** showed hCA I, K_i_ > 100 μM; hCA II, K_i_ > 100 μM; hCA IX, K_i_ = 9.2 μM; and hCA XII, K_i_ = 8.0 μM. In silico studies revealed key interaction residues of the molecule **71b** and confirmed the binding mode and stability of ligand interactions within the binding pockets of the enzymes hCA IX and hCA XII, which were overexpressed in hypoxic cancer cells.

The anticancer activity of coumarin–chalcone hybrids tested by Amin et al. (2013) [84] against a human colon cancer cell line (HCT-116) showed compound **79o** (Scheme 16, Figure 6) to exhibit excellent anticancer activity, with an IC_50_ of 0.01 μM. The investigation was extended for the inhibition of PI3K (P110α/p85α), and the IC_50_ value was found to be 50.78 μM.

Compound **93k** (Scheme 22, Figure 6) also exhibited strong activity, with IC_50_ values against H4IIE (IC_50_ = 1.62 ± 0.57 μM), HepG2 (IC_50_ = 8.212 ± 1.14 μM), and CHO (IC_50_ = 21.490 ± 3.22 μM) [89].

**Figure 7:**
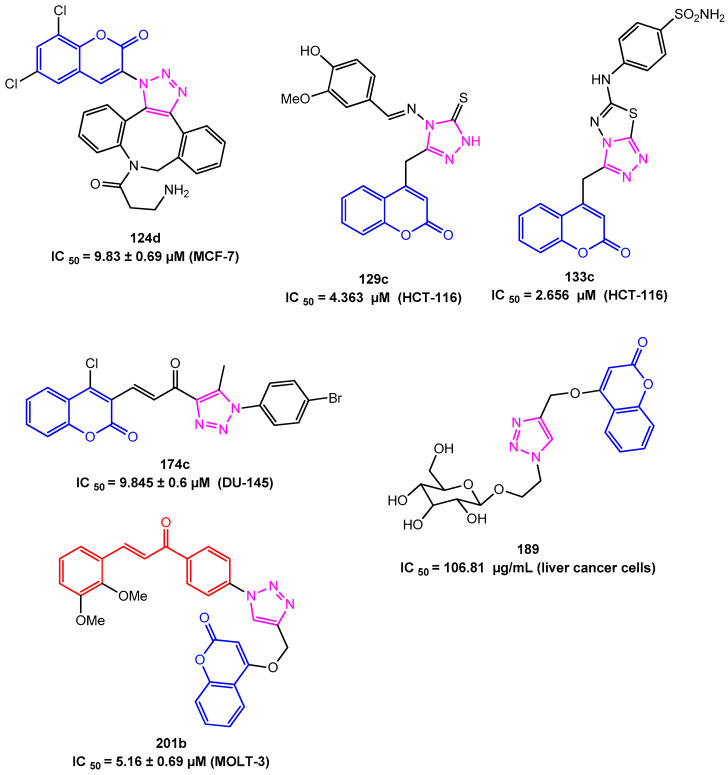
More examples of coumarin-based compounds as anticancer agents.

Pavić et al. (2021) tested novel coumarin–chalcone hybrids in vitro against human cancer cell lines. Compound **107b** (Scheme 26, Figure 7) was the most active, with IC_50_ values of HepG-2 = 19.5 ± 1.5 μM; SW620 = 20.8 ± 0.4 μM; HCT116 = 4.7 ± 0.6 μM; MCF-7 = 6.9 ± 0.7 μM; and Hek293T > 50 μM [93].

A new class of coumarin–triazole compounds developed by Sinha et al. (2016) were evaluated for anticancer activity via an MTT bioassay. Compound **124d** (Scheme 29, Figure 7) showed the highest potency and selectivity in all the tested cancer cell lines, with IC_50_ values of HeLa = 17.5 ± 1.22 μM, MCF-7 = 9.83 ± 0.69 μM, and MRC-5 = 185.22 ± 1.65 μM [96]. 

Similarly, a series of coumarin–triazole derivatives were synthesized and evaluated as anticancer agents against a human colorectal cancer cell line by Al-Wahaibi et al. (2018) [97]. The compounds **129c** and **133c** (Scheme 30, Figure 7) exhibited marked cytotoxic activity, with IC_50_ values of 4.363 μM and 2.656 μM, respectively. 

Among several compounds developed by Vagish et al. (2021), coumarin–chalcone **174c** (Scheme 39, Figure 7) was found to show higher potency against the PC-3, DU-145, and COX-2 cell lines with IC_50_ values of 10.538 ± 0.3 μM, 9.845 ± 0.6 μM, and 32.450 ± 0.18 μM, respectively [106]. 

The anticancer activity of coumarin–1,2,3–triazole–glucoside hybrids was evaluated against liver cancer using an MTT assay liver cancer [109]. The highest activity was demonstrated by compound **189** (Scheme 42, Figure 7) with an IC_50_ value of 106.81 μg/mL.

Among the chalcone–coumarin hybrids developed by Pingaew et al. (2014), compound **201b** (Scheme 45, Figure 6) possessed the highest anticancer activity against HuCCA-1, HepG2, A549, and MOLT-3, with IC_50_ values of 11.13 ± 0.40 μM, 15.70 ± 2.00 μM, 31.40 ± 1.00 μM, and 5.16 ± 0.69 μM, respectively [112]. Molecular docking was also performed to investigate the binding of the ligands to potential targets. The highest binding energy was found to be −9.6 kcal/mol.

### 3.4. Antidiabetic Activity

**Figure 8:**
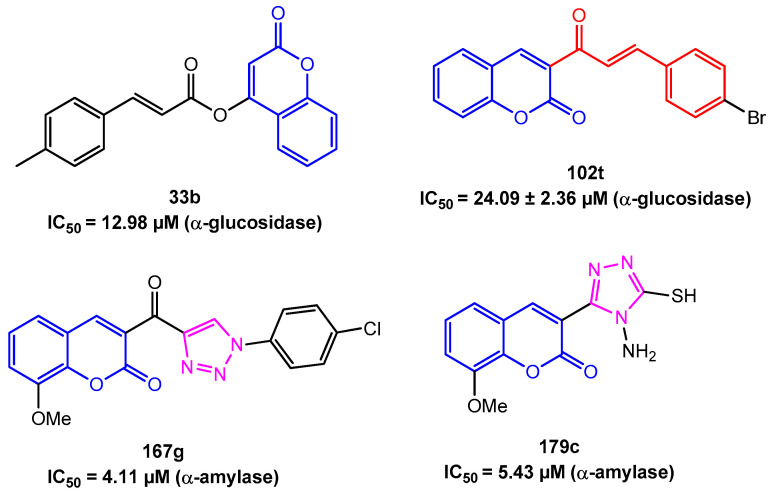
Coumarin-based compounds as antidiabetic agents.

Among the coumarin derivatives developed by Xu et al. (2020), compound **33b** (Scheme 7, Figure 8) showed the strongest α-glucosidase inhibition with an IC_50_ value of 12.98 μM [74].

Among the coumarin–chalcone derivatives developed by Hu et al. (2022) as α-glucosidase inhibitors, compound **102t** (Scheme 25, Figure 8) displayed the highest IC_50_ value of 24.09 ± 2.36 μM [92].

Coumarin-tethered 1,2,3-triazole analogues developed by Vagish et al. (2021) showed variable in vitro *α*-amylase inhibition activities [106]. Compound **174g** (Scheme 39, Figure 8) showed potent α-amylase inhibition with an IC_50_ value of 4.11 μM. The molecular docking of compound **174g** using α-amylase showed docking score of −5.60 kcal/mol.

The antidiabetic activity screening of coumarin–triazole hybrids developed by Basappa et al. (2020) revealed compound **179c** (Scheme 40, Figure 8) to exhibit potent inhibition of α-amylase with an IC_50_ value of 5.43 μM. [107].

### 3.5. Anti-Cholinesterase Activity

**Figure 9:**
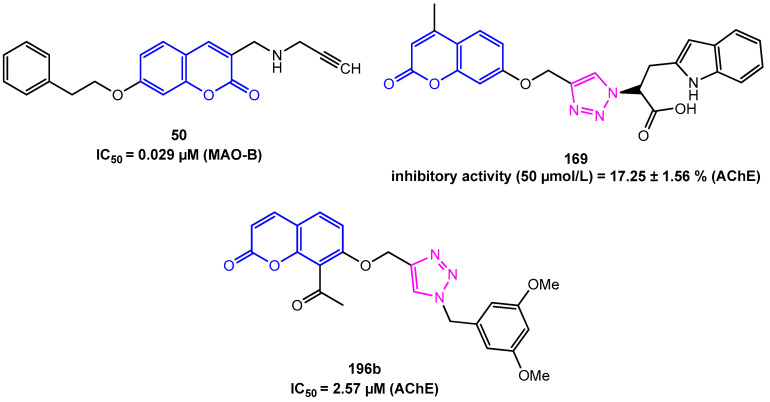
Coumarin-based compounds as anti-cholinesterase.

Mzezewa et al. (2021) examined 3,7-substituted coumarin derivatives as inhibitors of monoamine oxidase (MAO) and cholinesterase enzymes [78]. Compound **50** (Scheme 11, Figure 9) inhibited MAO-A (IC_50_ = 3.86 μM), MAO-B (IC_50_ = 0.029 μM), AChE (IC_50_ = 108 μM), and BuChE (IC_50_ = 105 μM). In silico studies using the SwissADME tool predicted that compound **50** could have acceptable pharmacokinetic and drug-likeness properties.

The coumarin–triazole hybrids developed by De Sousa et al. (2021) were also evaluated for anti-cholinesterase activity via the inhibition of acetylcholinesterase [105]. Compound **169** (Scheme 38, Figure 9) showed an inhibitory activity of 17.25 ± 1.56% at 50 μmol/L.

The coumarin–triazole hybrids developed by Sharma and Bharate (2023) [111] were screened for their inhibition of ChEs and BACE-1. The most active compound, **196b** (Scheme 44, Figure 9), inhibited acetylcholinesterase (AChE), butyrylcholinesterase (BChE), and β-secretase-1 (BACE-1) with IC_50_ values of 2.57, 3.26, and 10.65 μM, respectively.

### 3.6. Anti-Inflammatory Activity

**Figure 10:**
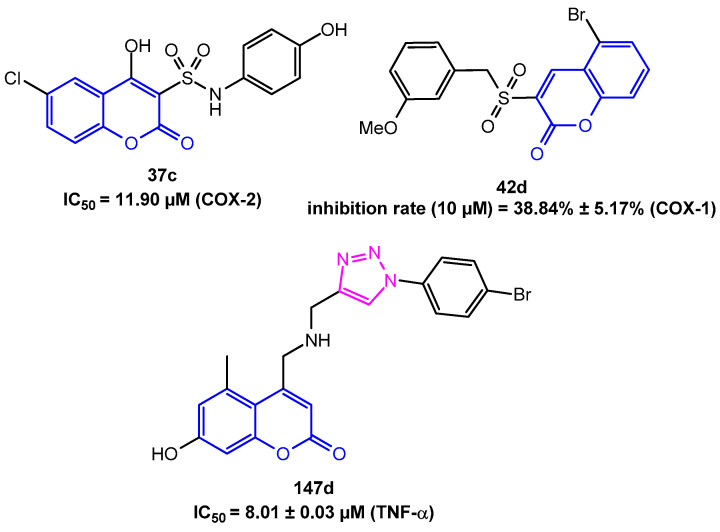
Coumarin-based compounds as anti-inflammatory agents.

Alshibl et al. (2020) developed coumarin derivatives and examined their anti-inflammatory activity [75]. Compound **37c** (Scheme 8, Figure 10) showed a proteinase-inhibitory activity of 41.69 ± 2.83%. It also showed 9.45% inhibition (at 2.5 mg/kg) for a prophylactic anti-inflammatory effect on formaldehyde-induced rat paw edema. It exhibited IC_50_ values of 15.40 μM for COX-1 and 11.90 μM for COX-2. Molecular docking predicted binding affinity between compound **37c** and human COX-2 isozyme at −7.3 kcal/mol.

Coumarin derivatives such as compound **42d** (Scheme 9, Figure 10) suppressed the release of TNF-α and exhibited favorable inhibitory activity at a concentration of 10 μM against COX-1 (38.84 ± 5.17% inhibition) and COX-2 (35.71 ± 4.01% inhibition) [76]. Furthermore, at a dose of 20 mg/kg, compound **42d** effectively reduced ear swelling with 41.43% inhibition.

Coumarin–triazole derivatives assessed in vitro by estimating the pro-inflammatory cytokine TNF-α in an LPS-stimulated U937 cell line showed compound **147d** (Scheme 33, Figure 10) to significantly inhibit TNF-α at a 10 μM concentration, with 62.03 ± 0.28% inhibition and an IC_50_ value of 8.01 ± 0.03 μM [100].

### 3.7. Miscellaneous Activity

**Figure 11:**
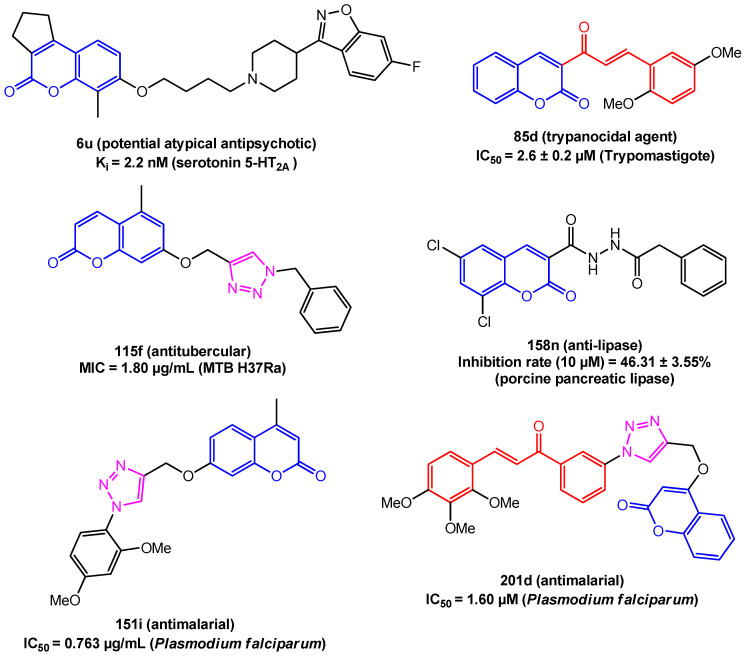
Other bioactivities of coumarin-based compounds.

Chen et al. (2014) synthesized new coumarin derivatives as potential atypical antipsychotics [68]. Compound **6u** (Scheme 1, Figure 11) showed a high affinity for the dopamine D_2_ and D_3_ receptors and serotonin 5-HT_1A_ and 5-HT_2A_ receptors, with low affinity for the H_1_ receptor (D_2_, K_i_ = 12.7 nM; D_3_, K_i_ = 13.6 nM; 5-HT_1A_, K_i_ = 7.8 nM; 5-HT_2A_, K_i_ = 2.2 nM; H_1_, K_i_ = 1825.3 nM).

Coumarin–chalcone derivatives developed by Rodríguez (2015) were tested against the epimastigote, trypomastigote, and amastigote stages of the *T. cruzi* parasite [86]. The results indicated that compound **85d** (Scheme 19, Figure 11) was the most potent trypanocidal agent, with IC_50_ values at different parasite stages as follows: Epimastigote = 46.8 ± 3.7 μM; Trypomastigote = 2.6 ± 0.2 μM; and Amastigote = 2.9 ± 0.1 μM. The IC_50_ values in mammalian cell lines were as follows: RAW 264.7 = 6.1 ± 0.5 μM; VERO = 56.8 ± 5.4 μM.

The potential antitubercular activity of coumarin–triazole derivatives was investigated against MTB H37Ra (ATCC 25177) by Shaikh et al. (2016) [95]. Compound **115f** (Scheme 28, Figure 11) exhibited interesting and highly promising antitubercular activity (MIC = 1.80 μg/mL). Compound **115f** was reported to inhibit the DprE1 enzyme of MTB and has the most active binding mode in the active site of the DprE1 enzyme.

N. Yadav et al. (2018) successfully synthesized a series of novel coumarin–triazole derivatives as antiplasmodials [101]. The compounds were evaluated for in vitro antiplasmodial activity against a chloroquine-sensitive strain of *Plasmodium falciparum* (3D7). Compound **151i** (Scheme 34, Figure 11) was found to be the most active, with an IC_50_ value of 0.763 ± 0.0124 μg/mL.

A series of novel coumarin hydrazides were investigated against porcine pancreatic lipase by Yilmaz and Faiz (2018) [102]. Compound **158n** (Scheme 35, Figure 11) showed the highest activity among the synthesized compounds, with 46.31 ± 3.55% pancreatic lipase inhibition at 10 μM.

Pingaew et al. (2014) developed chalcone–coumarin hybrids and evaluated their antimalarial activity against *Plasmodium falciparum* [112]. Compound **201d** (Scheme 45, Figure 11) proved to be the most potent compound (IC_50_ = 1.60 μM). The results of an in-silico study with falcipain-2, a cysteine protease from *P. falciparum*, showed a binding energy of −8.1 kcal/mol.

## 4. Conclusions and Prospective

Herein, we reviewed recent trends (2013–2023) in the synthesis and bioactivity of coumarin, coumarin–chalcone molecular hybrids, and coumarin–triazole molecular hybrids. The chemistry to synthesize these compounds was enabled by several established reactions including Pechmann condensation, Claisen–Schmidt condensation, and azide–alkyne cycloaddition reactions. The synthesized compounds showed diversity in their structures in terms of substituent types, substitution pattern, and the fusion/attachment point to coumarin. The reactions proceeded in a few steps under mild conditions and usually gave good-to-excellent yields. A wide range of bioactivities including antioxidant, antimicrobial, anticancer, antidiabetic, and anti-cholinesterase activities were also reviewed. Although some structure–activity studies (SAR) have been presented in some cases, extensive SARs are lacking in the literature. Additionally, though significant progress was made in synthesis and bioactivity screening, the mechanism of action remains to be investigated in detail. Several questions need to be addressed: do the hybrids interact with the known receptors of one or both chromophores or do they target new receptors? How does the synergistic effect take place? An understanding of this mechanism is critical for optimizing the structures of the hybrids to deliver optimum synergistic effects. The development of molecular hybrids continues to be an active and dynamic field with potential future prospects in medicinal chemistry and health science. It is our hope that these compounds will be useful for the treatment of various diseases.
